# Tumor Microenvironment‐Responsive Nanoreactor Induces Disulfidptosis in Pancreatic Cancer via Metabolic Interference and Redox Catalysis

**DOI:** 10.1002/advs.76146

**Published:** 2026-06-15

**Authors:** Rui Fu, Qing Li, Guanzhong Zhao, Qingyan Gao, Huantong Chen, Khemayanto Hidayat, Jiaying Xu, Liqiang Qin, Chunhong Hu, Yu Chong, Su Hu

**Affiliations:** ^1^ Department of Radiology The First Affiliated Hospital of Soochow University Suzhou China; ^2^ State Key Laboratory of Radiation Medicine and Protection, School of Radiation Medicine and Protection, Collaborative Innovation Center of Radiological Medicine of Jiangsu Higher Education Institutions Soochow University Suzhou China; ^3^ Department of Interventional Radiology Renmin Hospital of Wuhan University Wuhan China; ^4^ Department of Nutrition and Food Hygiene, School of Public Health Soochow University Suzhou China

**Keywords:** disulfidptosis, GLUT1, metabolism, redox, SLC7A11/xCT

## Abstract

Pancreatic cancer exhibits extensive metabolic reprogramming that supports rapid progression and therapeutic resistance, making metabolic vulnerabilities attractive targets for intervention. Here, a tumor microenvironment‐responsive nanoreactor (Pht@HMnO_2_‐HA) is designed to induce disulfidptosis in pancreatic cancer through coordinated metabolic interference and redox catalysis. Following CD44‐mediated tumor targeting and cellular internalization, the nanoreactor's responsive self‐optimization within the tumor microenvironment enables localized release of phloretin, suppressing glucose uptake and pentose phosphate pathway activity and thereby limiting intracellular reducing‐power generation. In parallel, the nanoreactor consumes intracellular glutathione and amplifies oxidative stress via MnO_2_‐mediated redox reactions, thereby depleting antioxidant defenses. Together, these processes impose reducing‐power deprivation and disrupt redox homeostasis, leading to cystine accumulation, disulfide stress, actin cytoskeleton collapse, and disulfidptosis in pancreatic cancer cells. Moreover, degradation‐associated Mn^2^^+^ release provides activatable T1‐weighted MRI contrast, enabling noninvasive visualization of intratumoral nanoreactor activation and therapeutic progression. Collectively, this work establishes a theranostic nanoreactor that exploits coupled metabolic and redox vulnerabilities to induce disulfidptosis, offering a mechanistically grounded strategy for precision therapy in pancreatic cancer.

## Graphical abstract

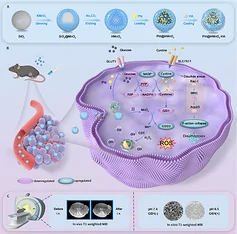

## 1 Introduction

Pancreatic cancer is among the most lethal solid tumors, with a 5‐year survival rate of less than 10% [1, 2], and its incidence and mortality continue to rise worldwide [3, 4]. In addition to its well‐known genetic and molecular heterogeneity, pancreatic cancer is characterized by a highly active and adaptable metabolic program [5] that drives rapid progression and therapeutic resistance [6]. Accordingly, increasing attention has been directed toward metabolic vulnerabilities as potential targets for intervention.

A prominent feature of pancreatic cancer metabolism is its strong dependence on glucose. Tumor cells maintain high glycolytic flux even under aerobic conditions, a phenotype known as the Warburg effect [7, 8]. This dependence is largely sustained by glucose transporter 1 (GLUT1), which mediates increased glucose uptake [9]. Beyond its role in ATP generation, glucose metabolism provides carbon input to the pentose phosphate pathway (PPP), which generates nicotinamide adenine dinucleotide phosphate (NADPH) and supports intracellular redox balance [10, 11]. At the same time, accelerated metabolic activity imposes a substantial oxidative burden. To counteract this stress, pancreatic cancer cells frequently upregulate the cystine/glutamate antiporter (SLC7A11/xCT) [12], thereby enhancing cystine uptake for glutathione (GSH) synthesis. The intracellular reduction of cystine to cysteine is highly dependent on NADPH, linking antioxidant defense directly to glucose‐derived reducing power [13]. As a result, glucose metabolism and redox homeostasis are tightly coupled through NADPH production and consumption.

This coupling creates a potential metabolic vulnerability. Inhibition of GLUT1 reduces glucose uptake and suppresses PPP activity, thereby reducing NADPH availability. Phloretin (Pht), a naturally occurring small‐molecule inhibitor of GLUT1, has been shown to reduce glucose transport and limit NADPH supply [14]. Under such conditions, cells with high SLC7A11 expression fail to efficiently reduce imported cystine, resulting in intracellular disulfide accumulation, cytoskeletal destabilization, and the induction of a distinct form of cell death termed disulfidptosis [15, 16]. In parallel, reduced cysteine availability compromises GSH biosynthesis, further intensifying intracellular oxidative stress. Accumulating evidence indicates that disulfide stress and oxidative stress are not independent phenomena but instead reinforce each other, collectively driving cell death [17].

Despite this mechanistic rationale, therapeutic strategies based solely on GLUT1 inhibition remain insufficient. Residual intracellular GSH pools and adaptive antioxidant responses, such as NRF2 activation, can partially buffer redox stress and limit cytotoxicity [18, 19, 20]. In addition, phloretin exhibits poor aqueous solubility and limited bioavailability [21], and its pharmacokinetic behavior and tumor accumulation are suboptimal [22]. Dense stromal barriers further restrict intratumoral penetration and uniform drug distribution [23], making it challenging to achieve sustained disruption of redox homeostasis in vivo. Therefore, an effective strategy should not only inhibit glucose‐dependent reducing‐power generation but also synchronously weaken intracellular antioxidant buffering and amplify redox stress within the tumor microenvironment.

To overcome these limitations, hollow mesoporous manganese dioxide (HMnO_2_) was explored as a redox‐adaptive platform capable of both efficient Pht delivery and direct intracellular GSH depletion [24]. In the tumor microenvironment, HMnO_2_ undergoes reductive degradation, consuming GSH and releasing Mn^2^^+^ ions. The released Mn^2^^+^ can catalyze hydroxyl radical (•OH) generation, thereby amplifying oxidative stress [25] and potentially enhancing disulfide accumulation. This dual redox activity offers an opportunity to reinforce metabolic interference while ultimately exhausting antioxidant defenses.

On this basis, we designed a tumor microenvironment‐responsive nanoreactor composed of hyaluronic acid (HA)‐coated, Pht‐loaded hollow mesoporous manganese dioxide nanoparticles (Pht@HMnO_2_‐HA). HA enables CD44‐mediated tumor targeting and facilitates stromal penetration [26, 27]. Tumor microenvironment–responsive degradation triggers Pht release, leading to GLUT1 inhibition, suppressed PPP activity, and reduced NADPH production [28, 29]. Simultaneously, HMnO_2_‐mediated GSH depletion and Mn^2^^+^‐catalyzed reactive oxygen species generation cooperatively amplify redox stress [30]. In addition, Mn^2^^+^ provides T1‐weighted magnetic resonance imaging (T1‐MRI) contrast signal for noninvasive visualization of therapeutic activation [31, 32, 33]. Unlike previously reported approaches that mainly rely on single‐pathway metabolic restriction or general oxidative amplification, our design integrates tumor‐responsive drug release, reducing‐power deprivation, antioxidant exhaustion, and activatable T1‐MRI within a single nanoreactor. Together, this integrated system exploits the coupled metabolic‐redox vulnerability of pancreatic cancer cells to enable imaging‐guided disulfidptosis induction.

## 2 Results and Discussion

### 2.1 PAAD Exhibits SLC7A11/GLUT1 Upregulation and Supports Targeting GLUT1 With Pht

**FIGURE 1:**
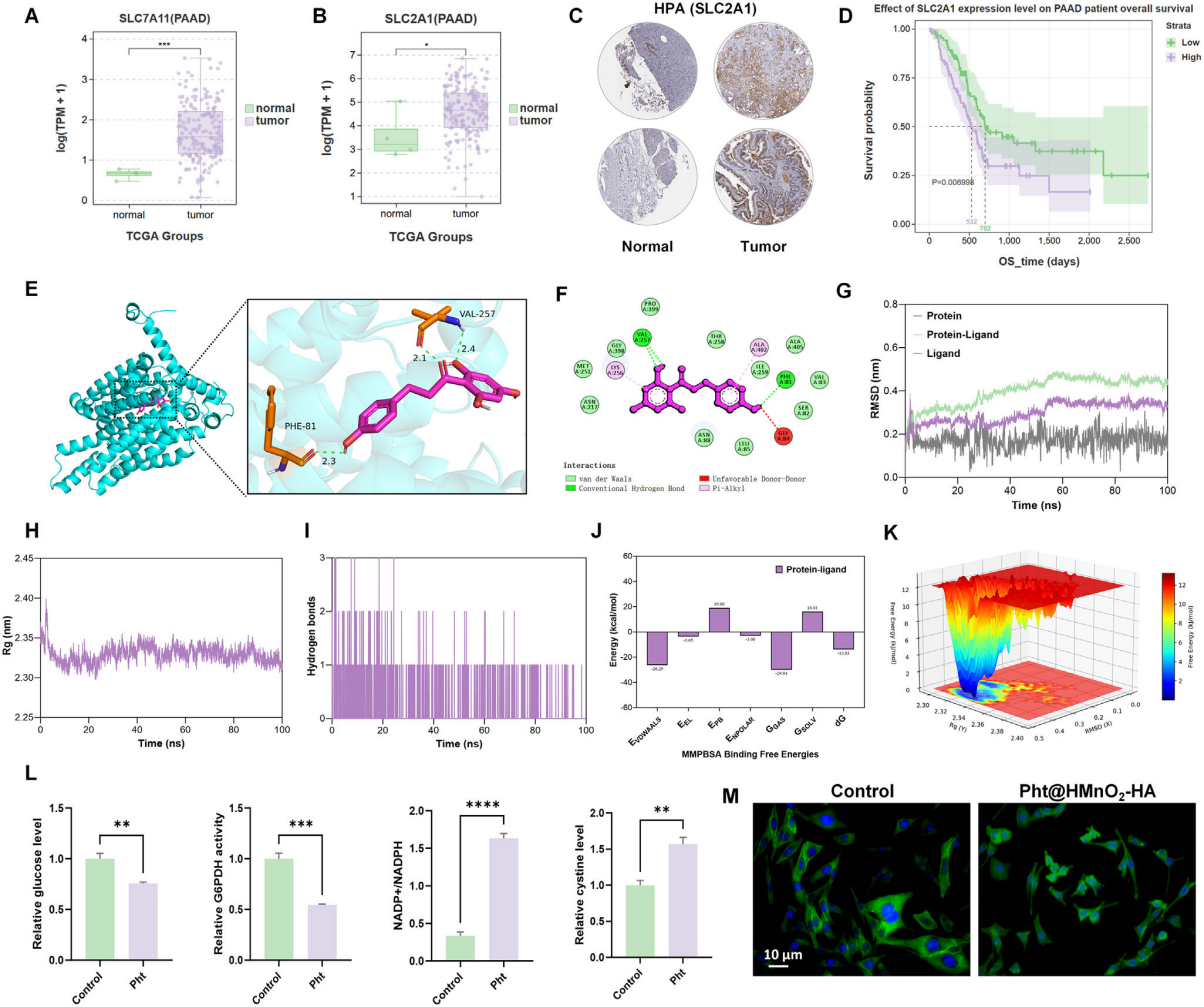
Clinical relevance of SLC7A11/SLC2A1 (GLUT1) in PAAD and multi‐level evaluation of Pht–GLUT1 inhibition. (A) SLC7A11 expression in normal pancreatic tissue and PAAD samples from TCGA. (B) SLC2A1 (GLUT1) expression in normal pancreatic tissue and PAAD samples from TCGA. (C) Immunohistochemical staining of SLC2A1 (GLUT1) in normal pancreatic tissue and PAAD samples from the Human Protein Atlas (HPA). (D) Kaplan–Meier overall survival analysis of patients with PAAD, stratified by SLC2A1 expression. (E) Molecular docking pose of Pht bound to GLUT1 (magenta, Pht; cyan ribbon structure, GLUT1). (F) Two‐dimensional interaction map of key residues involved in Pht‐GLUT1 interactions. (G) Root Mean Square Deviation (RMSD) of the protein, protein–ligand complex, and ligand during a 100 ns molecular dynamics simulation. (H) Radius of gyration (Rg) of the protein–ligand complex during the molecular dynamics simulation. (I) Number of hydrogen bonds between Pht and GLUT1 during the molecular dynamics simulation. (J) MM‐PBSA binding free energy of the protein–ligand complex. (K) Free energy landscape (FEL) of the protein‐ligand complex derived from MD trajectories. (L) Quantification of intracellular glucose level, G6PDH activity, NADP^+^/NADPH ratio, and intracellular cystine level following Pht treatment. (M) Fluorescence images of F‐actin (green) and nuclei (DAPI, blue). Scale bar: 10 µm. Data are presented as mean ± standard deviation (SD). Statistical significance is indicated: *p < 0.05, **p < 0.01, ***p < 0.001, and ****p < 0.0001.

To determine whether pancreatic adenocarcinoma (PAAD) exhibits a metabolic context compatible with disulfidptosis vulnerability, we first examined the expression of SLC7A11 using TCGA datasets. SLC7A11 expression was significantly upregulated in PAAD compared with normal pancreatic tissue (Figure 1A). A pan‐cancer analysis further showed that SLC7A11 upregulation was a common feature across multiple tumor types, including PAAD (Figure S1). In PAAD, higher SLC7A11 expression showed a trend toward poorer overall survival, although this did not reach statistical significance (Figure S2). This observation suggests that SLC7A11 may primarily reflect a permissive metabolic state rather than serving as an independent prognostic factor.

Given that disulfidptosis has been reported to occur preferentially in SLC7A11‐high cells under conditions of limited reducing capacity [34, 35, 36], we next assessed whether PAAD simultaneously upregulates an upstream glucose‐supply pathway that sustains cellular reducing power. TCGA analysis revealed marked overexpression of SLC2A1 (the gene encoding GLUT1) in PAAD relative to normal tissue (Figure 1B), a finding consistent with its broad upregulation across cancer types (Figure S3). At the protein level, immunohistochemical (IHC) data from the Human Protein Atlas showed stronger GLUT1 staining in PAAD than in normal pancreas (Figure 1C). Kaplan–Meier analysis further indicated that high SLC2A1 expression was significantly associated with worse overall survival (Figure 1D). Together, these results indicate that PAAD is characterized by concurrent elevation of SLC7A11‐mediated cystine demand and GLUT1‐driven glucose uptake, supporting GLUT1 as a rational upstream target for perturbing glucose‐dependent reducing capacity in this disease.

To evaluate the feasibility of pharmacologically targeting GLUT1, we selected Pht as a representative GLUT1 inhibitor and examined its interaction with GLUT1 using molecular docking and molecular dynamics (MD) simulations. Docking analysis showed that Pht occupies the transmembrane cavity of GLUT1 with a defined binding pose (Figure 1E). Interaction mapping identified specific contacts between Pht and key residues, including Phe81 and Val257 (Figure 1F).

Subsequent 100 ns MD simulations demonstrated stabilization of the protein, ligand, and complex, as reflected by convergent RMSD trajectories (Figure 1G). The radius of gyration remained relatively constant throughout the simulation (Figure 1H), and hydrogen‐bond interactions between Pht and GLUT1 persisted over time (Figure 1I), indicating sustained binding. Consistently, MM‐PBSA analysis yielded an overall negative binding free energy (Figure 1J), and the free energy landscape analysis revealed dominant low‐energy basins populated during the trajectory (Figure 1K). Additional MD metrics, including limited residue‐level fluctuations (RMSF), stabilized solvent‐accessible surface area (SASA), and distinct per‐residue energetic contributions to binding, further supported the stability of the Pht‐GLUT1 complex (Figure S4).

Having established stable GLUT1 engagement by Pht in silico, we then examined whether GLUT1 inhibition produced the expected metabolic consequences at the cellular level. Pht treatment reduced intracellular glucose levels and glucose‐6‐phosphate dehydrogenase (G6PDH) activity, increased the NADP^+^/NADPH ratio, and led to intracellular cystine accumulation (Figure 1L). These changes are consistent with impaired glucose uptake, suppressed PPP activity, and diminished availability of reducing power. Finally, since disulfidptosis is characterized by disruption of the actin cytoskeleton [37], fluorescence microscopy showed that Pht‐treated cells exhibited disorganization and fragmentation of F‐actin structures compared with controls (Figure 1M). These results establish GLUT1 as an actionable upstream metabolic node in PAAD and provide a mechanistic basis for exploiting Pht‐mediated GLUT1 inhibition in a disulfidptosis‐prone metabolic context.

### 2.2 Synthesis and Characterization of Pht@HMnO_2_‐HA

**SCHEME 1:**
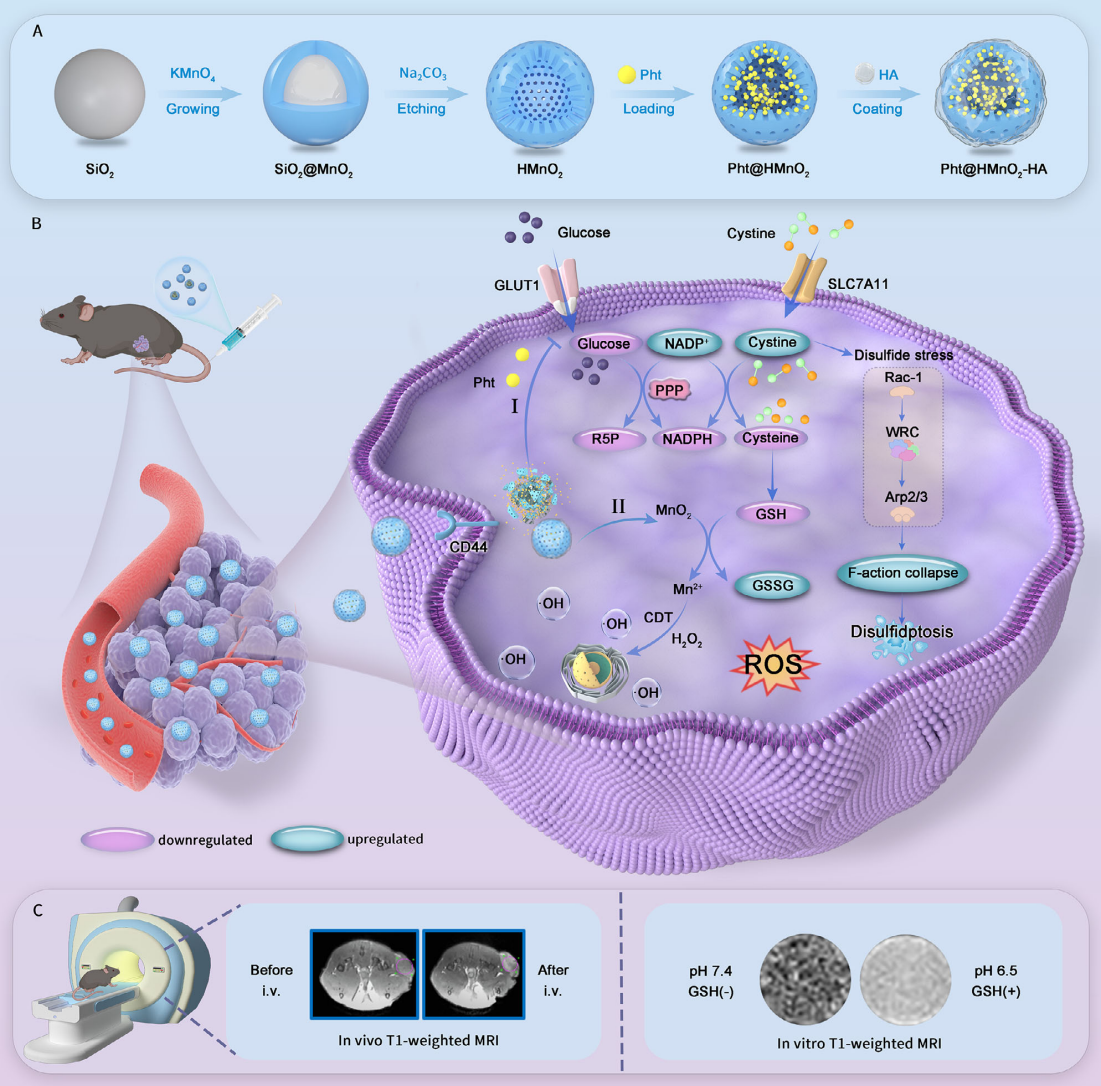
Schematic illustration of a tumor microenvironment‐responsive nanoreactor for imaging‐guided induction of disulfidptosis in pancreatic cancer via metabolic interference and redox catalysis. (A) Stepwise fabrication of Pht@HMnO_2_‐HA, including MnO_2_ growth on a SiO_2_ template, template removal to form hollow mesoporous MnO_2_, Pht loading, and subsequent surface functionalization with HA. (B) After tumor accumulation and cellular internalization via CD44‐mediated recognition, the nanoreactor exerts a dual mode of action: Pht‐mediated suppression of glucose uptake reduces intracellular reducing‐power generation, while tumor‐microenvironment‐responsive MnO_2_ degradation disrupts redox buffering capacity, enhances disulfide stress, and induces actin cytoskeleton collapse, collectively leading to disulfidptosis in pancreatic cancer cells. (C) Tumor microenvironment‐triggered MnO_2_ degradation releases Mn^2^^+^ ions, generating T1‐weighted MRI contrast and enabling noninvasive visualization of intratumoral nanoreactor activation.

As shown in Scheme 1A, the Pht@HMnO_2_‐HA nanoreactor was constructed through a stepwise assembly strategy that integrates Pht loading with an outer HA layer for CD44‐mediated tumor‐selective delivery. In this design, Pht inhibits GLUT1 and reduces NADPH production. HMnO_2_ serves as a tumor‐microenvironment‐responsive carrier that depletes GSH and enhances oxidative stress. HA improves stability and facilitates CD44‐mediated tumor targeting. Molecular docking analysis indicated that HA can be accommodated within the CD44 binding pocket and engage representative residues through stabilizing interactions, as reflected by the binding conformation, close‐up interaction view, and two‐dimensional interaction map (Figure S5).

Monodisperse SiO_2_ nanospheres were first synthesized as templates using a base‐catalyzed Stöber method with TEOS. A MnO_2_ shell was subsequently deposited onto the SiO_2_ surface through an interfacial reaction in KMnO_4_ solution, yielding SiO_2_@MnO_2_ core–shell nanoparticles. Selective etching of the SiO_2_ core with Na_2_CO_3_ produced hollow mesoporous MnO_2_ (HMnO_2_) nanospheres. To facilitate subsequent experiments, both empty carrier nanoparticles (HMnO_2_‐HA) and drug‐loaded nanoparticles (Pht@HMnO_2_‐HA) were prepared. For the carrier control, HA was covalently conjugated onto the surface of HMnO_2_ through surface amination followed by EDC/NHS‐mediated amidation to obtain HMnO_2_‐HA. For the drug‐loaded formulation, Pht was first loaded into the hollow mesoporous HMnO_2_ structure via physical adsorption to form Pht@HMnO_2_, after which HA was grafted to obtain Pht@HMnO_2_‐HA. Optimization of the Pht‐to‐HMnO_2_ feed ratio revealed a clear dependence of loading capacity and encapsulation efficiency on the mass ratio (Figure S11A); a 4:1 ratio was therefore adopted for all subsequent preparations. This stepwise strategy enabled efficient Pht encapsulation while introducing an HA shell that improves colloidal stability and provides CD44‐recognition capability.

**FIGURE 2:**
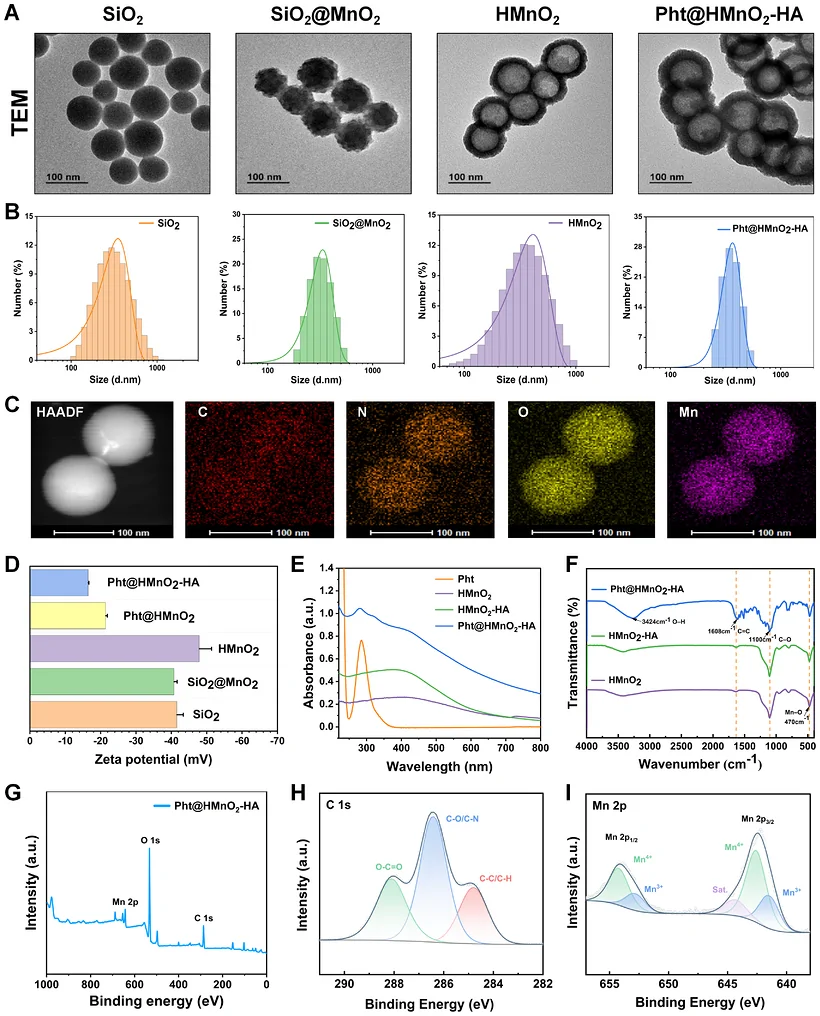
Synthesis and characterization of Pht@HMnO_2_‐HA. (A) TEM images of SiO_2_, SiO_2_@MnO_2_, HMnO_2_, and Pht@HMnO_2_‐HA. (B) Particle size distribution of SiO_2_, SiO_2_@MnO_2_, HMnO_2_, and Pht@HMnO_2_‐HA measured by DLS. (C) HAADF‐STEM image and corresponding elemental mapping of C, N, O, and Mn for Pht@HMnO_2_‐HA. (D) Zeta potentials of SiO_2_, SiO_2_@MnO_2_, HMnO_2_, Pht@HMnO_2_, and Pht@HMnO_2_‐HA. (E) UV‐vis absorption spectra of Pht, HMnO_2_, HMnO_2_‐HA, and Pht@HMnO_2_‐HA. (F) FTIR spectra of HMnO_2_, HMnO_2_‐HA, and Pht@HMnO_2_‐HA. (G–I) XPS spectra of Pht@HMnO_2_‐HA: (G) survey spectrum, (H) C 1s region, and (I) Mn 2p region.

The successful construction of the Pht@HMnO_2_‐HA nanoreactor was confirmed through comprehensive characterization of morphology, size distribution, elemental composition, surface charge, and chemical states. Transmission electron microscopy (TEM) images illustrated the structural evolution during synthesis (Figure 2A and Figure S6A,B). The initial SiO_2_ templates appeared as uniform solid spheres with smooth surfaces. After MnO_2_ deposition, a continuous and dense coating layer was observed, forming well‐defined SiO_2_@MnO_2_ core‐shell structures. Following selective removal of the SiO_2_ core, the resulting HMnO_2_ nanospheres exhibited a distinct internal cavity and uniform shell thickness, consistent with a hollow mesoporous architecture. The final Pht@HMnO_2_‐HA nanoreactor retained this spherical hollow morphology, indicating that the subsequent drug loading and polymer modification did not compromise the structural integrity of the carrier.

Dynamic light scattering (DLS) measurements showed a progressive increase in average hydrodynamic diameter from ∼327 nm for SiO_2_ to ∼338 nm for SiO_2_@MnO_2_, ∼362 nm for HMnO_2_, and ∼377 nm for Pht@HMnO_2_‐HA (Figure 2B). Notably, DLS reflects the hydrodynamic diameter in aqueous dispersion, which is typically larger than the dry‐state size observed by TEM. Moreover, the hydrodynamic diameter of Pht@HMnO_2_‐HA remained essentially unchanged over 7 days in H_2_O, PBS, and DMEM, confirming its colloidal stability under physiologically relevant conditions (Figure S7).

High‐angle annular dark‐field scanning transmission electron microscopy (HAADF‐STEM) elemental mapping revealed homogeneous distributions of C, N, O, and Mn throughout Pht@HMnO_2_‐HA, consistent with uniform coating of the inorganic HMnO_2_ framework with the organic components (Figure 2C). The corresponding energy‐dispersive X‐ray spectroscopy (EDS) spectrum showed distinct Mn, O, and C signals (Figure S6C). Zeta potential analysis further reflected the stepwise assembly process, with the surface charge evolving systematically during synthesis and ultimately yielding a stable negative potential for the final Pht@HMnO_2_‐HA nanoreactor, consistent with successful HA functionalization (Figure 2D).

Ultraviolet‐visible (UV‐vis) spectroscopy confirmed successful drug loading, as a characteristic absorption peak of Pht at ∼285 nm [38] was present in the Pht@HMnO_2_‐HA spectrum but absent in both HMnO_2_ and HMnO_2_‐HA (Figure 2E). Fourier‐transform infrared spectroscopy (FTIR) further provided additional evidence for surface modification and drug incorporation. HMnO_2_ exhibited a characteristic Mn─O stretching vibration at ∼470 cm^−^^1^ [39]. After HA conjugation, new bands corresponding to O─H stretching (∼3400 cm^−^^1^) and carboxyl C═O stretching (∼1630–1650 cm^−^^1^) appeared [40]. The Pht@HMnO_2_‐HA spectrum further displayed characteristic Pht signals, including aromatic C═C skeletal vibrations (∼1510–1600 cm^−^^1^) and phenolic C─O stretching (∼1200–1260 cm^−^^1^) [41], which were absent in the control samples (Figure 2F), confirming successful drug incorporation.

X‐ray photoelectron spectroscopy (XPS) was employed to analyze the surface chemical composition and oxidation states. As shown in Figure S8 and Figure 2G, survey spectra of HMnO_2_ and Pht@HMnO_2_‐HA showed characteristic Mn and O signals, confirming the successful construction of the MnO_2_ framework after functionalization. High‐resolution C 1s spectrum of Pht@HMnO_2_‐HA (Figure 2H) was deconvoluted into C–C/C–H (∼284.8 eV), C–O (∼286.5 eV), and O–C═O (∼288.5 eV) components. Compared with HMnO_2_ (Figure S9), Pht@HMnO_2_‐HA exhibited increased relative contributions from oxygenated carbon species, consistent with surface enrichment of HA and Pht and enhanced surface hydrophilicity. Mn 2p spectra displayed characteristic Mn 2p_3_/_2_ and Mn 2p_1_/_2_ peaks at ∼642 and ∼653 eV, respectively (Figure 2I), with binding energies comparable to those of HMnO_2_ (Figure S10) and accompanied by a weak satellite feature, suggesting that the dominant MnO_2_‐related oxidation state characteristics were largely preserved after functionalization [42, 43]. Overall, these results support the successful construction of a structurally intact, colloidally stable, and chemically well‐defined Pht@HMnO_2_‐HA nanoreactor suitable for subsequent biological investigations.

### 2.3 Functional Validation of the Tumor Microenvironment‐Responsive Pht@HMnO_2_‐HA Nanoreactor

Metabolic reprogramming in pancreatic cancer results in a tumor microenvironment with slightly acidic pH and elevated intratumoral GSH levels [44, 45]. In contrast, the circulation maintains near‐neutral pH and low plasma GSH [46]. This discrepancy provides a rationale for designing tumor‐selective drug delivery systems. We therefore examined the structural stability and responsiveness of Pht@HMnO_2_‐HA under conditions mimicking physiological and tumor environments.

**FIGURE 3:**
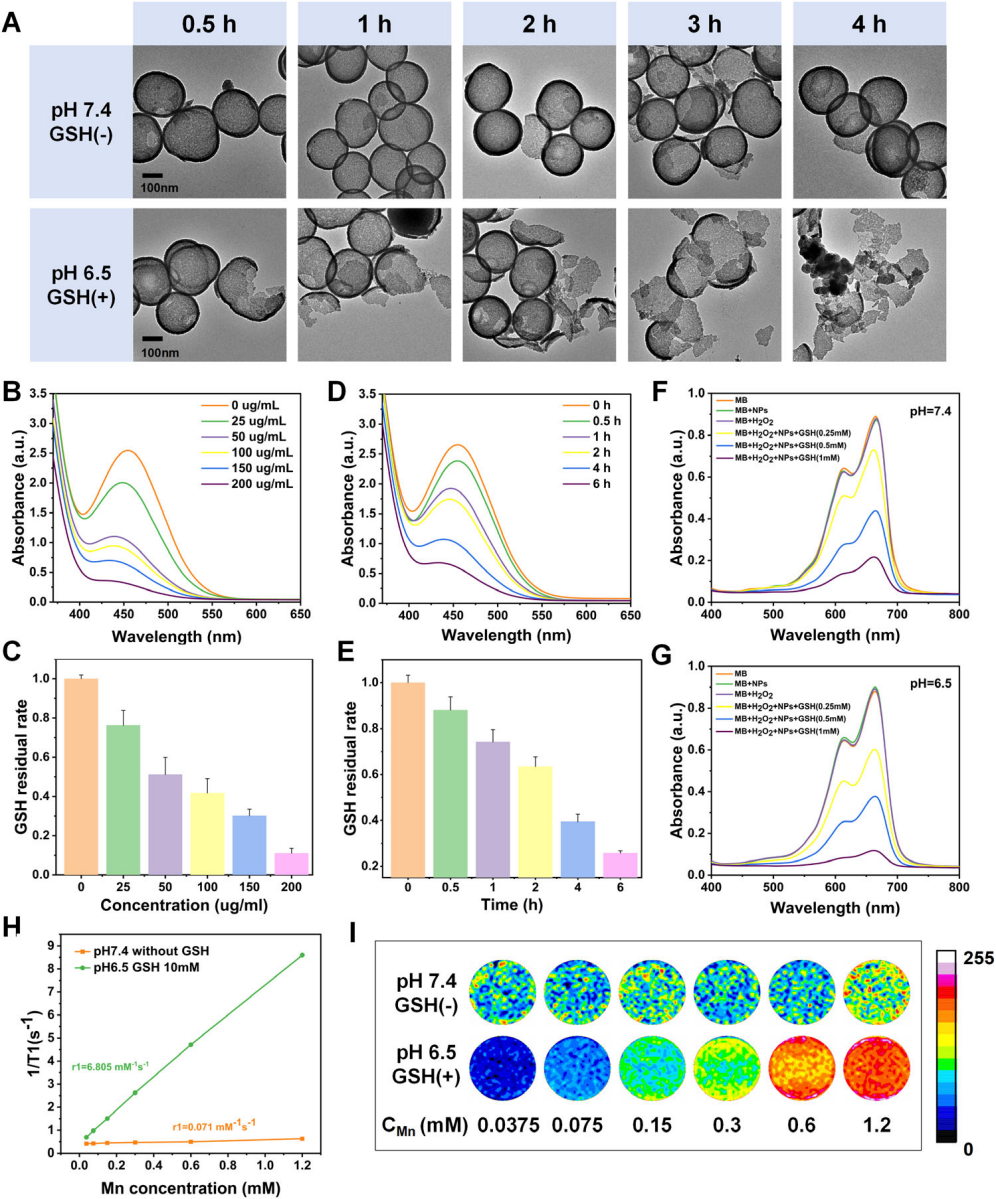
Functional evaluation of Pht@HMnO_2_‐HA. (A) TEM images of Pht@HMnO_2_‐HA after incubation under physiological conditions (pH 7.4, without GSH) and tumor‐mimicking conditions (pH 6.5, with GSH) for 0.5–4 h. (B) UV‐vis absorption spectra of reaction products formed between residual GSH and DTNB after treatment with different concentrations of Pht@HMnO_2_‐HA. (C) Quantification of residual GSH corresponding to (B). (D) UV‐vis absorption spectra of reaction products formed between residual GSH and DTNB after incubation with Pht@HMnO_2_‐HA for different time periods. (E) Quantification of residual GSH corresponding to (D). (F, G) UV‐vis absorption spectra of methylene blue (MB) under different conditions at pH 7.4 (F) and pH 6.5 (G). (H) Longitudinal relaxivity (r_1_) of Pht@HMnO_2_‐HA at different Mn^2^^+^ concentrations under pH 7.4 (without GSH) and pH 6.5 (with GSH). (I) T1‐weighted MR images of Pht@HMnO_2_‐HA solutions at different Mn^2^^+^ concentrations in the absence or presence of GSH.

As shown in Figure 3A, Pht@HMnO_2_‐HA maintained its spherical morphology after 4 h of incubation under simulated physiological conditions (pH 7.4, absence of GSH). In contrast, under tumor‐mimicking conditions (pH 6.5, 5 mM GSH), the nanoparticles exhibited time‐dependent structural degradation, characterized by progressive shell thinning and eventual disintegration. These observations indicate that the nanoreactor undergoes stimuli‐responsive structural breakdown. Consistent with this behavior, the in vitro release behavior of Pht from Pht@HMnO_2_‐HA was further evaluated. Pht release was minimal and slow under physiological conditions (pH 7.4, absence of GSH), whereas a significantly accelerated release profile with a higher cumulative amount was observed under tumor‐mimicking conditions (pH 6.5, with GSH) (Figure S11B), demonstrating microenvironment‐triggered drug release. These results confirm that Pht can be efficiently retained under physiological conditions but rapidly released in a tumor‐mimicking microenvironment, thereby supporting the microenvironment‐responsive design of the platform.

We next evaluated the ability of Pht@HMnO_2_‐HA to deplete GSH, a key component of cellular redox homeostasis. Using the DTNB assay, we found that residual GSH levels decreased significantly with increasing nanoparticle concentrations (25–200 µg mL^−1^) after 2 h of incubation (Figure 3B,C). At a fixed GSH concentration, GSH depletion increased progressively with incubation time (0.5–6 h) (Figure 3D,E). These results confirm efficient GSH consumption by the nanoreactor. Concurrently, the degradation of the MnO_2_ shell under acidic and reductive conditions releases Mn^2^^+^ ions, which can catalyze Fenton‐like reactions in the presence of H_2_O_2_ to generate ·OH [25]. •OH production was assessed using methylene blue (MB) as a probe. The characteristic MB absorption peak at ∼660–670 nm remained stable at pH 7.4. In contrast, in the presence of H_2_O_2_, nanoparticles, and GSH, the MB signal decreased, with more pronounced attenuation at higher GSH concentrations (Figure 3F). This effect was further enhanced at pH 6.5, where the MB peak was nearly quenched at elevated GSH levels (Figure 3G), indicating amplified ·OH generation under combined acidic and reductive conditions and supporting chemodynamic therapy (CDT) activity.

In addition to redox modulation, the release of Mn^2^^+^ ions endows the system with MRI capability. Longitudinal relaxivity (r_1_) measurements showed significantly higher r_1_ values under tumor‐mimicking conditions (pH 6.5, 5 mM GSH), reaching 6.805 mM^−^^1^·s^−^^1^, compared with physiological conditions (Figure 3H). Consistently, T_1_‐weighted MR images showed concentration‐dependent signal enhancement exclusively in GSH‐rich environments (Figure 3I and Figure S12), confirming the activatable MRI capability of the nanoreactor.

Overall, these solution‐level validations demonstrate that Pht@HMnO_2_‐HA undergoes tumor microenvironment–responsive degradation, enables on‐demand drug release, efficiently depletes GSH, and amplifies oxidative stress generation. In the context of the SLC7A11‐high metabolic phenotype of pancreatic cancer, such coordinated reducing‐power exhaustion and oxidative stress are expected to perturb cystine metabolism and facilitate disulfidptosis. These findings establish a mechanistic foundation for subsequent cellular and in vivo investigations of the antitumor activity of Pht@HMnO_2_‐HA.

### 2.4 In Vitro Antitumor Activity of Pht@HMnO_2_‐HA Under Energy Restriction and Oxidative Stress

Based on its tumor microenvironment–responsive degradation and redox‐modulating properties, the in vitro antitumor activity of Pht@HMnO_2_‐HA was evaluated, with particular focus on cellular uptake, cytotoxicity, and the associated energy restriction and oxidative stress responses. The intracellular processes examined here are schematically summarized in Scheme 1B.

**FIGURE 4:**
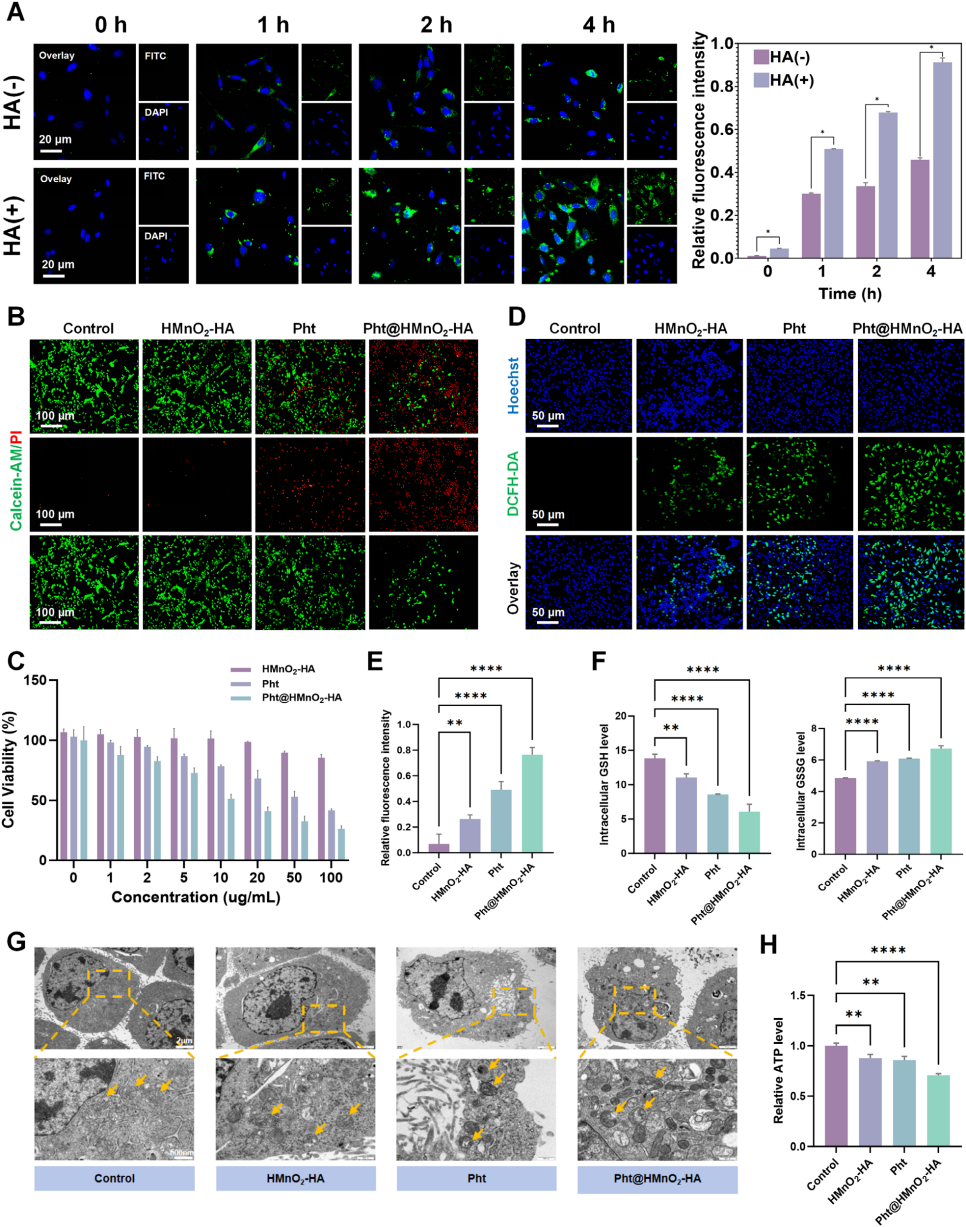
In vitro biological evaluation of Pht@HMnO_2_‐HA. (A) Confocal fluorescence images showing cellular uptake of Pht@HMnO_2_‐HA at different incubation times with or without HA coating, and corresponding quantification of relative fluorescence intensity (RFI). (B) Live/dead staining of cells after different treatments. (C) Cell viability following treatment with different concentrations of HMnO_2_‐HA, Pht, and Pht@HMnO_2_‐HA. (D) Intracellular ROS detection by DCFH‐DA staining under different treatments. (E) Quantification of intracellular ROS levels corresponding to (D). (F) Intracellular GSH and GSSG levels under different treatments. (G) TEM images showing ultrastructural changes in cells after different treatments. (H) Relative ATP levels of cells under different treatments. Data are presented as mean ± standard deviation (SD). Statistical significance is indicated: *p < 0.05, **p < 0.01, and **** *p* < 0.0001.

Cellular internalization of the nanoreactor was first examined by fluorescence imaging. As shown in Figure 4A, intracellular fluorescence intensity increased progressively with incubation time, indicating efficient cellular uptake. At the same time points, Pht@HMnO_2_‐HA exhibited stronger fluorescence than the non‐HA‐coated control, suggesting that HA modification enhances cellular internalization, likely through CD44‐mediated interactions [47].

The cytotoxic effects of different formulations were subsequently assessed. Live/dead staining showed that cells in the control and HMnO_2_‐HA groups remained predominantly viable (green), whereas treatment with Pht induced moderate cell death (red). In contrast, Pht@HMnO_2_‐HA treatment resulted in a substantial increase in PI‐positive cells (red), indicating significantly enhanced cytotoxicity (Figure 4B). Consistent with these observations, CCK‐8 assays demonstrated that Pht@HMnO_2_‐HA inhibited cell proliferation more effectively than either Pht or HMnO_2_‐HA alone, displaying a clear concentration‐dependent effect (Figure 4C and Figure S13).

To investigate the mechanisms underlying the observed cytotoxicity, intracellular reactive oxygen species (ROS) levels were measured. Cells treated with Pht@HMnO_2_‐HA exhibited a markedly increased fluorescent signal (Figure 4D) and significantly elevated ROS levels compared with other treatment groups (Figure 4E). This increase exceeded that induced by HMnO_2_‐HA or Pht alone, consistent with the combined effects of Mn^2^^+^‐mediated ROS generation and antioxidant depletion. Concurrently, intracellular GSH levels were significantly reduced following Pht@HMnO_2_‐HA treatment, accompanied by a corresponding increase in oxidized glutathione (GSSG) (Figure 4F and Figure S14), indicating sustained consumption of reducing equivalents and compromised antioxidant capacity.

The resulting redox imbalance led to subcellular dysfunction [48, 49]. Lipid peroxidation probe staining revealed increased oxidized signals and decreased reduced signals in Pht@HMnO_2_‐HA‐treated cells, indicating membrane lipid peroxidation damage (Figure S15). JC‐1 staining further showed a shift from red aggregates to green monomers, reflecting a loss of mitochondrial membrane potential (ΔΨm) and impaired mitochondrial function (Figure S16). TEM analysis revealed extensive ultrastructural damage, including mitochondrial swelling, disrupted cristae, cytoplasmic vacuolization, and organelle disorganization in treated cells (Figure 4G), consistent with severe oxidative stress. Correspondingly, intracellular ATP levels were significantly reduced following Pht@HMnO_2_‐HA treatment (Figure 4H), indicating disrupted energy metabolism due to mitochondrial dysfunction.

### 2.5 In Vitro Induction of Disulfidptosis by Pht@HMnO_2_‐HA and Mechanistic Investigation

**FIGURE 5:**
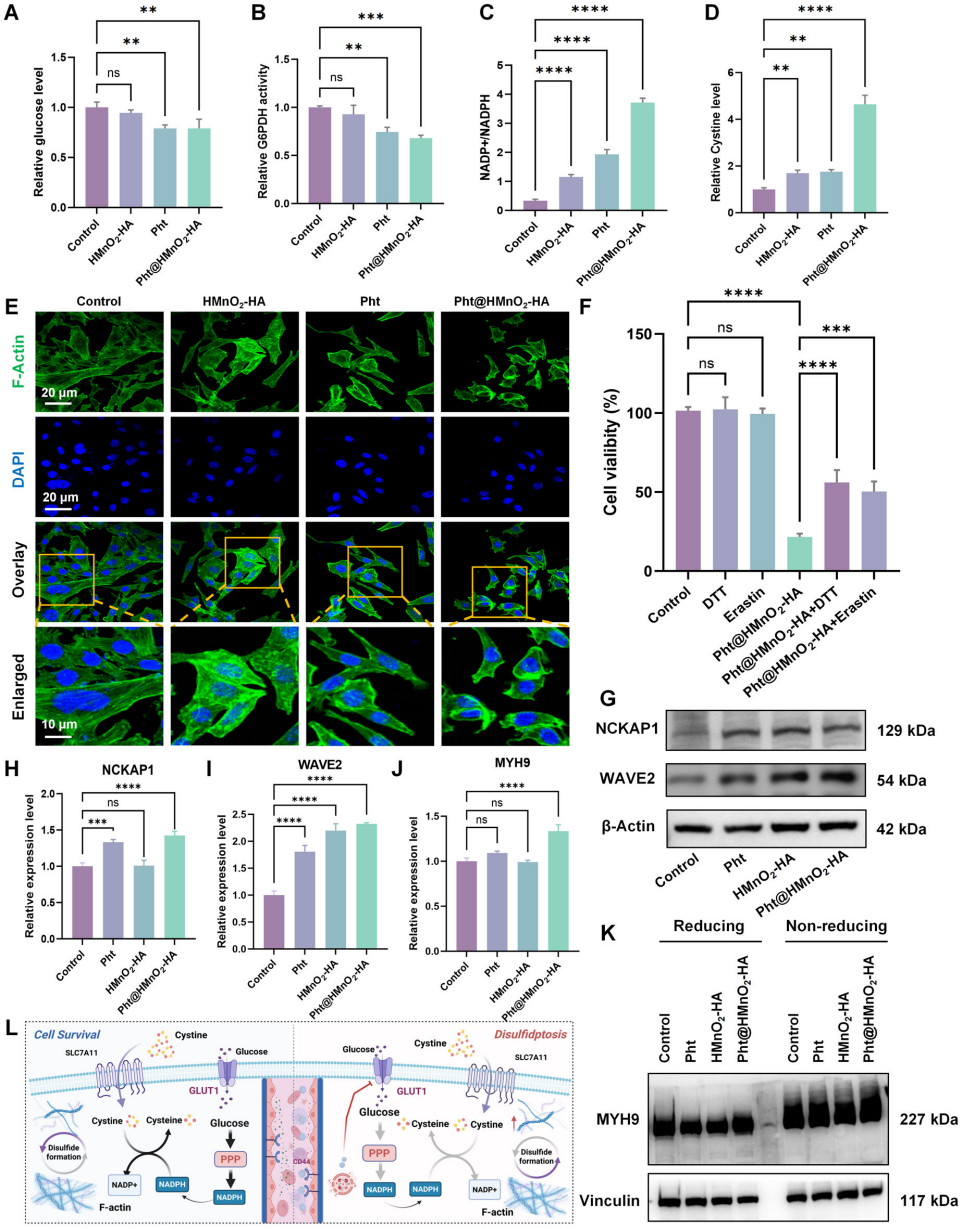
In vitro disulfidptosis induction mediated by Pht@HMnO_2_‐HA. (A) Relative intracellular glucose levels after different treatments. (B) G6PDH activity after different treatments. (C) Intracellular NADP^+^/NADPH ratio after different treatments. (D) Intracellular cystine levels after different treatments. (E) Confocal fluorescence images of cytoskeletal F‐actin (green) and nuclei (DAPI, blue) under different treatments. Enlarged images highlight changes in F‐actin organization. (F) Relative cell viability following treatment with Control, DTT, Erastin, Pht@HMnO_2_‐HA, Pht@HMnO_2_‐HA + DTT, and Pht@HMnO_2_‐HA + Erastin. (G) Western blot of NCKAP1 and WAVE2 protein expression under different treatments. (H,I) Quantitative analysis of NCKAP1 and WAVE2 expression levels after different treatments. (J) Quantitative analysis of MYH9 expression levels after different treatments under non‐reducing conditions. (K) Western blot of MYH9 under non‐reducing and reducing conditions. (L) Proposed mechanism of the disulfidptosis. Data are presented as mean ± standard deviation (SD). Statistical significance: *p < 0.05, **p < 0.01, ***p < 0.001, and ****p < 0.0001; ns, not significant.

Building on the cellular phenotypes described above, we next examined whether Pht@HMnO_2_‐HA induces disulfidptosis through coordinated disruption of glucose metabolism and redox homeostasis. Key metabolic parameters associated with reducing‐power generation were first evaluated. As shown in Figure 5A, intracellular glucose levels were significantly reduced following Pht@HMnO_2_‐HA treatment. In parallel, the activity of glucose‐6‐phosphate dehydrogenase (G6PDH), the rate‐limiting enzyme of the pentose phosphate pathway (PPP) [50], was markedly reduced in the Pht@HMnO_2_‐HA group compared with all other groups (Figure 5B), suggesting strong suppression of PPP flux. Consistently, the intracellular NADP^+^/NADPH ratio was significantly elevated after Pht@HMnO_2_‐HA treatment (Figure 5C), reflecting restricted NADPH production and a reduced supply of intracellular reducing equivalents.

Under these conditions of limited reducing power, intracellular cystine levels were examined. As shown in Figure 5D, cells treated with Pht@HMnO_2_‐HA showed pronounced cystine accumulation, whereas no significant changes were observed in the HMnO_2_‐HA or Pht groups relative to controls. These metabolic alterations were accompanied by marked cytoskeletal disruption. F‐actin fluorescence staining revealed that Pht@HMnO_2_‐HA treatment led to the collapse of the normally continuous and well‐organized F‐actin network, resulting in disordered cellular morphology. In contrast, control cells maintained intact, clearly defined stress fiber structures, whereas HMnO_2_‐HA or Pht treatment alone induced only minor cytoskeletal changes (Figure 5E).

To further examine the specificity of this cell death modality, cells were pretreated with dithiothreitol (DTT) or erastin prior to exposure to Pht@HMnO_2_‐HA. Both interventions significantly improved cell viability compared with Pht@HMnO_2_‐HA treatment alone (Figure 5F). The stronger protective effect of DTT is consistent with a disulfide‐dependent mechanism [51], while the partial rescue by erastin suggests functional involvement of cystine transport via SLC7A11 [52]. To further discriminate this process from other regulated cell death modalities, we performed an expanded rescue assay using inhibitors of apoptosis, necroptosis, autophagy‐related cell death, and ferroptosis. Pretreatment with Z‐VAD‐FMK, Nec‐1, CQ, Fer‐1, or DFO failed to markedly restore cell viability after Pht@HMnO_2_‐HA treatment, whereas the thiol‐reducing condition produced a pronounced rescue effect (Figure S17). These results further support that Pht@HMnO_2_‐HA‐induced cytotoxicity is mainly associated with disulfide stress rather than classical apoptosis, necroptosis, autophagy‐related cell death, or ferroptosis. At the protein level, Western blot analysis revealed altered expression of cytoskeleton‐associated regulators. Specifically, NCKAP1 and WAVE2 protein levels were increased following Pht@HMnO_2_‐HA treatment (Figure 5G), with quantitative analysis confirming significant upregulation compared with controls (Figure 5H–I). Analysis of MYH9 under reducing (RE) and non‐reducing (NR) conditions revealed migration patterns. Under NR conditions, MYH9 displayed slower migration, smearing, and high‐molecular‐weight aggregates in the Pht@HMnO_2_‐HA group, whereas normal migration was restored under RE conditions. Quantification under NR conditions further showed an increased MYH9 signal in high–molecular‐weight regions after Pht@HMnO_2_‐HA treatment (Figure 5J,K). Collectively, these results indicate that Pht@HMnO_2_‐HA promotes disulfide‐related protein aggregation, leading to cytoskeletal destabilization and disulfide stress–associated cell death, as schematically illustrated in Figure 5L.

**FIGURE 6:**
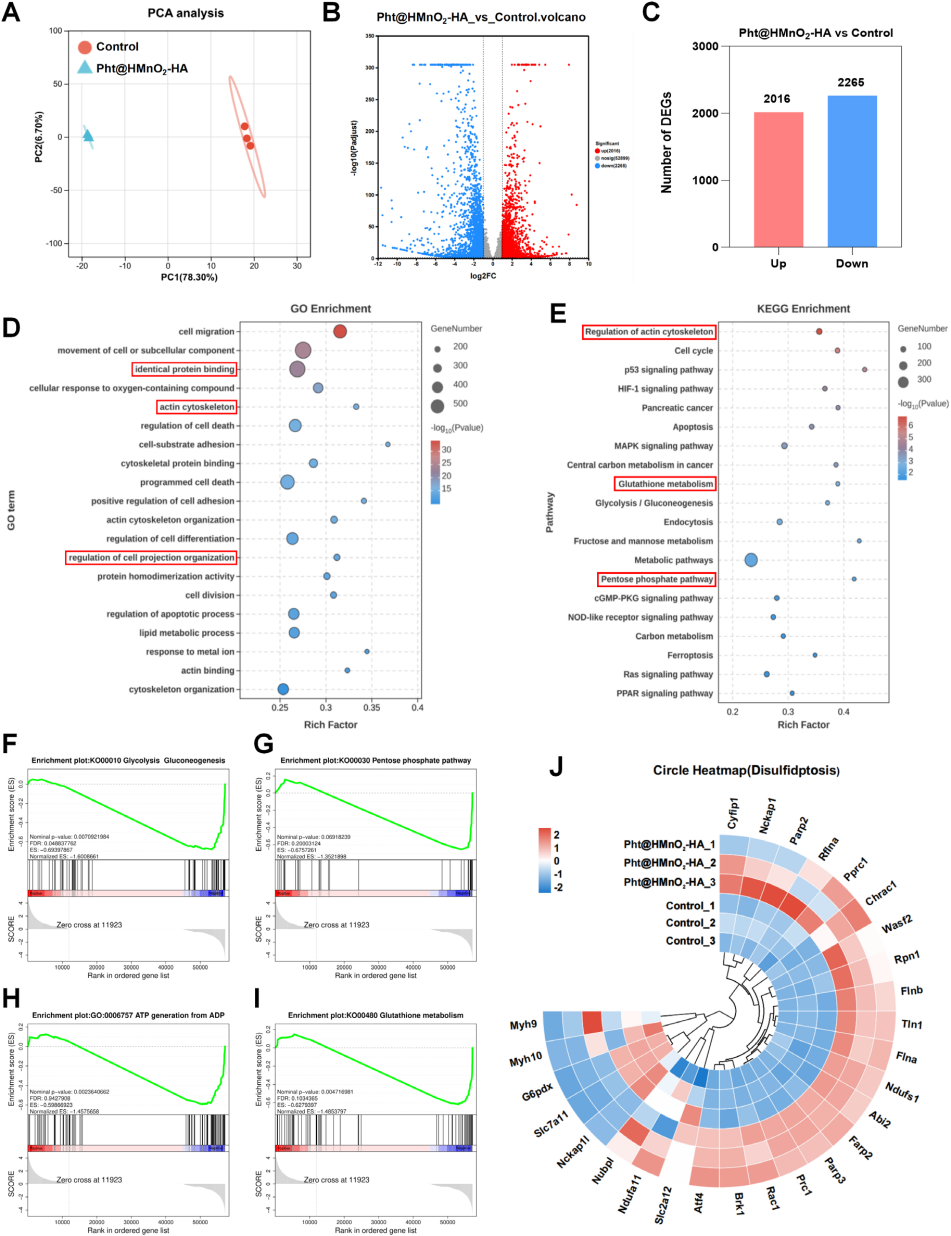
Transcriptomic analysis between Pht@HMnO_2_‐HA and Control groups. (A) PCA analysis of transcriptome profiles. (B) Volcano plot of differentially expressed genes (DEGs). (C) Number of upregulated and downregulated DEGs. (D) GO enrichment analysis of DEGs. (E) KEGG enrichment analysis of DEGs. (F–I) GSEA enrichment plots of representative pathways. (J) circular heatmap of representative cytoskeleton, metabolism, and disulfidptosis‐related genes.

To further elucidate the molecular basis of these effects at the transcriptional level, RNA sequencing was performed on cells treated with Pht@HMnO_2_‐HA and control cells. PCA showed clear separation between groups with tight intra‐group clustering (Figure 6A). Differential expression analysis revealed extensive transcriptional reprogramming in Pht@HMnO_2_‐HA‐treated cells (Figure 6B), with 2016 genes upregulated, and 2265 genes downregulated (Figure 6C). Integrated GO analyses indicated that these DEGs were predominantly associated with actin cytoskeleton regulation and metabolism–redox processes, including actin organization, oxidative stress responses, and cell death regulation (Figure 6D and Figure S18). KEGG pathway analysis further highlighted enrichment in pathways related to actin cytoskeleton regulation, the pentose phosphate pathway, and glutathione metabolism (Figure 6E and Figure S19), while Reactome analysis showed consistent perturbations in signal transduction, metabolic regulation, and cytoskeletal organization (Figure S20).

GSEA based on ranked transcriptomic profiles revealed coordinated suppression of glucose metabolism in the Pht@HMnO_2_‐HA group (Figure S21). More specifically, Glycolysis/gluconeogenesis and the pentose phosphate pathway were negatively enriched (Figure 6F,G and Figure S22A,B), accompanied by negative enrichment of ATP generation from ADP pathway (Figure 6H). Gene sets associated with glutathione biosynthesis and antioxidant activity were also negatively enriched (Figure 6I and Figure S22C), suggesting reduced transcriptional support for redox buffering. In contrast, apoptosis‐related gene signatures showed only modest positive enrichment (Figure S22D). A circular heatmap summarized the expression patterns of representative genes involved in cytoskeletal regulation, metabolism, and disulfidptosis across samples (Figure 6J).

Taken together, these metabolic, biochemical, and transcriptomic results collectively indicate that Pht@HMnO_2_‐HA induces extensive disruption of glucose metabolism, reducing‐power generation, antioxidant defense, and cytoskeletal integrity. The convergence of these alterations is consistent with a mechanism whereby coordinated metabolic and redox imbalance may promote disulfidptosis in pancreatic cancer cells.

### 2.6 In Vivo Antitumor Efficacy, Imaging Performance, and Biosafety Evaluation of Pht@HMnO_2_‐HA

**FIGURE 7:**
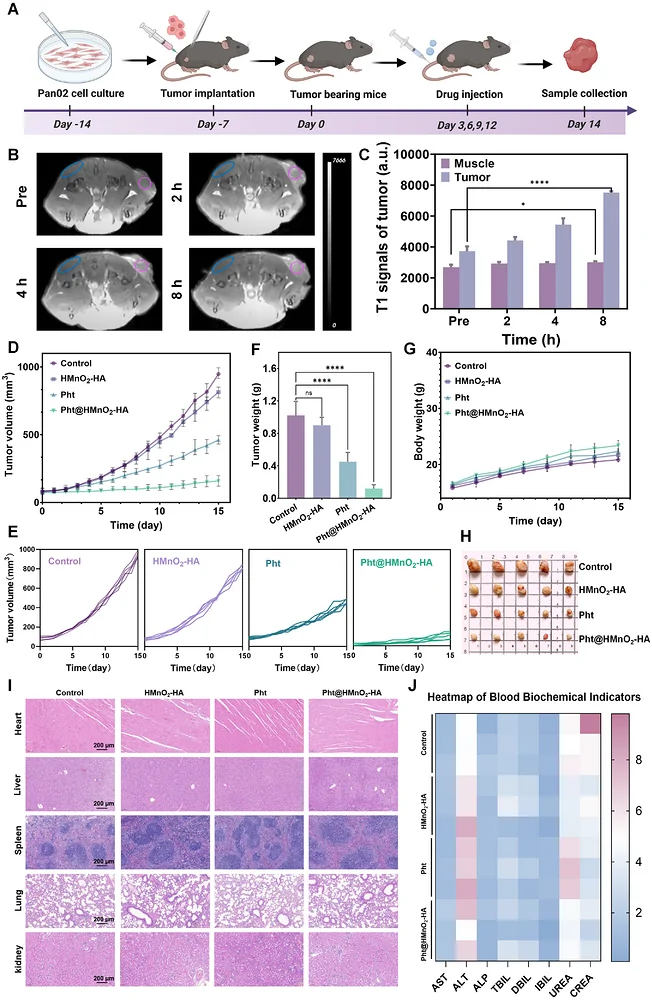
In vivo antitumor efficacy, imaging performance, and biosafety evaluation of Pht@HMnO_2_‐HA. (A) Schematic illustration of the treatment schedule in tumor‐bearing mice. (B) Representative T1‐weighted MR images acquired before (Pre) and at 2, 4, and 8 h after intravenous injection of Pht@HMnO_2_‐HA; tumor and muscle regions are indicated. (C) Quantification of T1 signal intensity in tumor and muscle regions at different time points after injection. (D) Tumor growth curves after different treatments. (E) Individual tumor growth curves after different treatments. (F) Tumor weights after different treatments. (G) Body weight changes during the treatment period. (H) Representative images of excised tumors after different treatments. (I) H&E staining of heart, liver, spleen, lung, and kidney. (J) Heatmap of blood biochemical parameters for different treatment groups. Data are presented as mean ± standard deviation (SD). Statistical significance: *p < 0.05, ****p < 0.0001; ns, not significant.

Following validation of the in vitro activity of Pht@HMnO_2_‐HA, the imaging performance in vivo, antitumor efficacy, and biosafety were evaluated in Panc02 tumor–bearing C57BL/6 mice according to the treatment schedule shown in Figure 7A. T1‐weighted MRI revealed a time‐dependent signal enhancement within the tumor region after intravenous administration of Pht@HMnO_2_‐HA. The signal became increasingly pronounced at 2–8 h post‐injection (Figure 7B). Quantitative analysis confirmed that the tumor T1‐weighted signal intensity increased markedly over time and remained consistently higher than that in muscle (Figure 7C), supporting tumor‐localized MR contrast activation.

Tumor volumes in the control and HMnO_2_‐HA groups increased rapidly, whereas free Pht showed a moderate inhibitory effect; in contrast, Pht@HMnO_2_‐HA consistently maintained the lowest tumor burden throughout the treatment window (Figure 7D). Individual tumor growth curves further showed that this inhibitory effect was consistent across mice within the group (Figure 7E). Terminal tumor weight measurements were in good agreement with the volume data, with the mean tumor mass in the Pht@HMnO_2_‐HA group reduced to ∼30% of that in the control group, corresponding to a tumor inhibition rate of approximately 70% (Figure 7F). Representative photographs of excised tumors visually corroborated these findings, showing markedly smaller tumors in the Pht@HMnO_2_‐HA group compared with other groups (Figure 7H).

Beyond its potent antitumor efficacy, we further performed a comprehensive evaluation of the in vivo biosafety of Pht@HMnO_2_‐HA. As shown in Figure 7G, body weights remained stable and comparable across all treatment groups. Histological examination of major organs (heart, liver, spleen, lung, kidney) showed no apparent histopathological abnormalities based on H&E staining (Figure 7I). Hemolysis assays showed that erythrocyte pellets remained intact and the supernatants were nearly clear at concentrations up to 200 µg mL^−1^. Although slight hemolysis was observed at a high concentration (400 µg mL^−1^), both HMnO_2_‐HA and Pht@HMnO_2_‐HA exhibited minimal hemolytic activity at the working concentration used for in vivo and in vitro experiments (100 µg mL^−1^), comparable to that of the PBS negative control (Figure S23). Blood biochemical analyses showed no significant differences in alanine aminotransferase (ALT), aspartate aminotransferase (AST), or alkaline phosphatase (ALP) among groups. Likewise, levels of total bilirubin (TBIL), direct bilirubin (DBIL), indirect bilirubin (IBIL), and renal function–related parameters did not exhibit abnormal elevations or group‐dependent changes (Figure 7J and Figure S24). Together, these data suggest the absence of overt hepatotoxicity or nephrotoxicity under the tested conditions and support good systemic tolerability of Pht@HMnO_2_‐HA.

Overall, these in vivo results demonstrate that Pht@HMnO_2_‐HA provides effective tumor‐targeted imaging, achieves sustained tumor growth suppression, and exhibits favorable biosafety, supporting its potential as an imaging‐guided therapeutic nanoplatform.

### 2.7 Mechanistic Investigation of Pht@HMnO_2_‐HA Induced Disulfidptosis In Vivo

**FIGURE 8:**
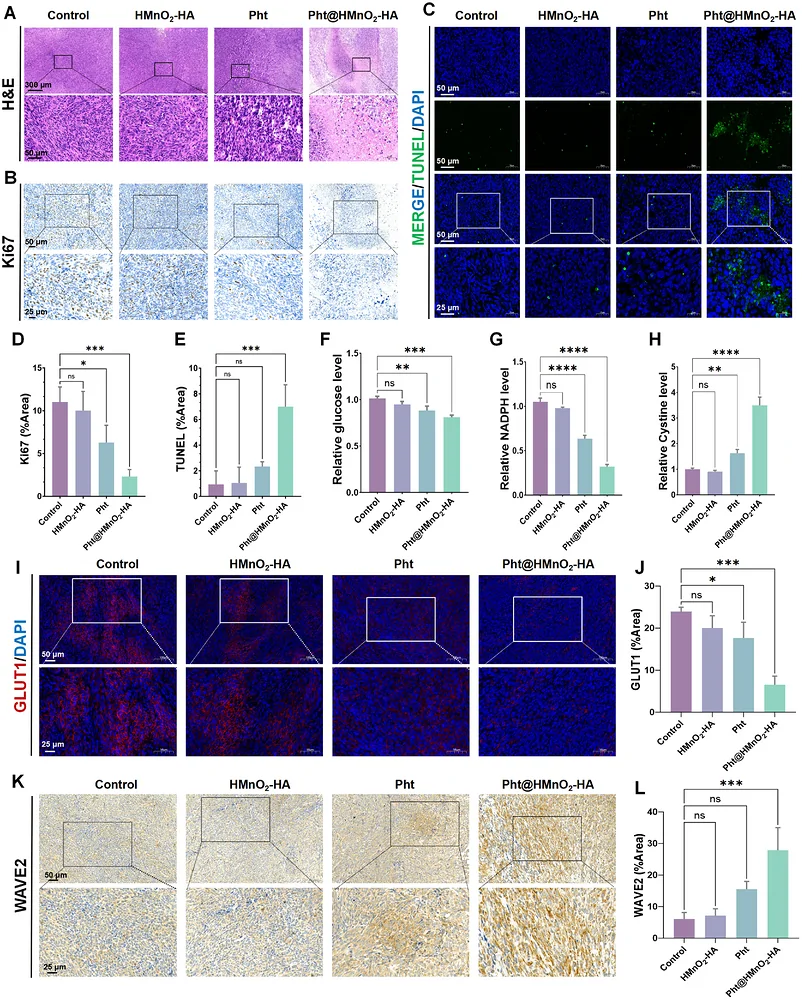
In vivo analysis of disulfidptosis‐related alterations in tumor tissues. (A) Representative H&E staining images of tumor sections from mice treated with Control, HMnO_2_‐HA, Pht, and Pht@HMnO_2_‐HA. (B) Immunohistochemical staining of Ki67 in tumor sections. (C) TUNEL staining of tumor sections. (D) Quantification of Ki67‐positive area in tumor sections. (E) Quantification of TUNEL‐positive area in tumor sections. (F) Relative intratumoral glucose levels measured in different treatment groups. (G) Relative intratumoral NADPH levels after different treatments. (H) Intratumoral cystine levels after different treatments. (I) Immunofluorescence staining of GLUT1 in tumor sections. (J) Quantification of GLUT1‐positive area in tumor sections. (K) Immunohistochemical staining of WAVE2 in tumor sections. (L) Quantification of WAVE2‐positive area in tumor sections. Data are presented as mean ± standard deviation (SD). Statistical significance: *p < 0.05, **p < 0.01, ***p < 0.001, and ****p < 0.0001; ns, not significant.

Consistent with the in vitro findings, tumor tissues collected after different treatments exhibited distinct histopathological and molecular changes in vivo. H&E staining showed that tumors from the control and HMnO_2_‐HA groups largely retained compact cellular architecture, whereas tumors treated with free Pht displayed only limited focal damage. In contrast, tumors from the Pht@HMnO_2_‐HA group exhibited extensive tissue disorganization with widespread necrotic and degenerative regions (Figure 8A). In parallel, immunohistochemical analysis of Ki67 revealed a marked reduction in proliferative activity in the Pht@HMnO_2_‐HA group (Figure 8B, D), while TUNEL staining demonstrated a pronounced increase in DNA fragmentation and cell death signals (Figure 8C, E). Together, these results indicate that Pht@HMnO_2_‐HA suppresses tumor proliferation and promotes tumor cell death in vivo.

To determine whether these tissue‐level effects were associated with metabolic alterations relevant to disulfidptosis vulnerability, we first assessed intratumoral metabolic and redox parameters. As shown in Figure 8F, intratumoral glucose levels were significantly reduced in the Pht@HMnO_2_‐HA group compared with other treatments. Consistently, tumor NADPH levels were markedly decreased (Figure 8G), indicating diminished reducing capacity in vivo. Under these conditions, intratumoral cystine accumulated to significantly higher levels in the Pht@HMnO_2_‐HA group (Figure 8H), consistent with limited cystine reduction and increased disulfide burden. We next examined glucose transporter expression within tumor tissues. Immunofluorescence staining revealed a significant decrease in GLUT1 expression following Pht@HMnO_2_‐HA treatment (Figure 8I), and quantitative analysis confirmed the lowest GLUT1‐positive area in this group (Figure 8J). Given that disulfidptosis is closely associated with cytoskeletal vulnerability under disulfide stress [53], we further evaluated the actin‐remodeling regulator WAVE2 [54]. Immunohistochemical staining demonstrated substantially increased WAVE2 expression in tumors treated with Pht@HMnO_2_‐HA, and quantitative analysis confirmed a significantly larger WAVE2‐positive area than in other groups (Figure 8K, L). To further support the in vivo cytoskeleton‐associated alterations, immunofluorescence staining of NCKAP1 and Rac1 was additionally performed in tumor tissues. As shown in Figure S25 and Figure S26, the expression trends of both NCKAP1 and Rac1 across the four groups were consistent with that of WAVE2, with the most pronounced changes observed in the Pht@HMnO_2_‐HA group. These findings further support the presence of disulfidptosis‐related cytoskeletal remodeling in vivo. In addition, immunofluorescence staining of GPX4 revealed attenuated expression in tumor tissues after Pht@HMnO_2_‐HA treatment (Figure S27), suggesting impaired antioxidant buffering capacity under this treatment condition. This result was used to reflect redox imbalance in the tumor microenvironment and was interpreted together with the disulfide stress–related evidence above. Collectively, these in vivo mechanistic data support that Pht@HMnO_2_‐HA induces a metabolic–redox environment conducive to disulfidptosis within tumor tissues, thereby reinforcing the mechanistic consistency between the in vitro and in vivo observations.

## 3 Conclusion

In summary, we developed a nanoreactor designed to respond to the tumor microenvironment and induce disulfidptosis in pancreatic cancer through coordinated metabolic interference and redox catalysis. Analysis of TCGA‐PAAD datasets showed concurrent upregulation of SLC7A11 and GLUT1, indicating a metabolic context that may be particularly sensitive to disulfidptosis when reducing capacity is limited and highlighting GLUT1 as a feasible upstream target. On this basis, the Pht@HMnO_2_‐HA system combines CD44‐mediated tumor targeting with microenvironment‐responsive degradation. This design decreases NADPH production derived from glucose, reduces intracellular glutathione levels, and increases oxidative stress within tumors. The resulting reduction in intracellular reducing capacity, reflected by an elevated NADP^+^/NADPH ratio, is accompanied by cystine accumulation and increased disulfide burden, leading to destabilization of the actin cytoskeleton and features consistent with disulfidptosis. Degradation of MnO_2_ also releases Mn^2^^+^, generating T1‐weighted MRI contrast and allowing noninvasive visualization of nanoreactor activation in tumors. Collectively, this work establishes an imaging‐guided therapeutic strategy that exploits metabolic and redox vulnerabilities in pancreatic cancer, providing a mechanistic basis for further development of precision nanomedicine approaches.

## Author Contributions

**Rui Fu**: conceptualization, writing – original draft, methodology, investigation. **Qing Li**: conceptualization, investigation, writing – original draft, methodology. **Chunhong Hu**: funding acquisition, writing – review and editing, project administration, supervision. **Liqiang Qin**: writing – review and editing. **Khemayanto Hidayat**: writing – review and editing. **Su Hu**: funding acquisition, writing – review and editing, project administration, supervision, resources. **Huantong Chen**: software, formal analysis. **Guanzhong Zhao**: writing – original draft, methodology, validation, visualization. **Jiaying Xu**: writing – review and editing. **Yu Chong**: funding acquisition, writing – review and editing, project administration, supervision, data curation. **Qingyan Gao**: software, formal analysis.

## Conflicts of Interest

The authors declare no conflicts of interest.

## Supporting information




**Supporting File**: advs76146‐sup‐0001‐SuppMat.docx.

## Data Availability

The data that support the findings of this study are available from the corresponding author upon reasonable request.
