# Supplementary material for: Tumor Microenvironment‐Responsive Nanoreactor Induces Disulfidptosis in Pancreatic Cancer via Metabolic Interference and Redox Catalysis
Source: Adv Sci (Weinh). 2026 Jun 15;13(51):e76146. doi: 10.1002/advs.76146 (PMC13336420; doi:10.1002/advs.76146)
Supplement: Supplementary file 1 [file ADVS-13-e76146-s001.docx]

**Supporting Information**

**Tumor Microenvironment-Responsive Nanoreactor Induces Disulfidptosis in Pancreatic Cancer via Metabolic Interference and Redox Catalysis**

*Rui Fu^#^, Qing Li^#^, Guanzhong Zhao, Qingyan Gao, Huantong Chen, Khemayanto Hidayat, Jiaying Xu, Liqiang Qin, Chunhong Hu*, Yu Chong***, Su Hu**

R. Fu, C. Hu, S. Hu

Department of Radiology, The First Affiliated Hospital of Soochow University, Suzhou, 215000, China

E-mail: huchunhong@suda.edu.cn; husu@suda.edu.cn

R. Fu, G. Zhao, J. Xu, Y. Chong

State Key Laboratory of Radiation Medicine and Protection, School of Radiation Medicine and Protection, Collaborative Innovation Center of Radiological Medicine of Jiangsu Higher Education Institutions, Soochow University, Suzhou, 215123, China

E-mail: chongyu@suda.edu.cn

K. Hidayat, L. Qin

Department of Nutrition and Food Hygiene, School of Public Health, Soochow University

199 Renai Road, Suzhou, Jiangsu 215123, China

Q. Li

Department of Interventional Radiology, Renmin Hospital of Wuhan University, Wuhan, China.

*^#^*These authors contributed equally to this work.

**Methods**

**Animal care**

Male C57BL/6 mice (6 weeks old) were purchased from GemPharmatech Co., Ltd. (Jiangsu, China) under license number 1100111084356. All animal procedures were approved by the Ethics Committee of Soochow University (Approval No. SUDA20251013A03). The number of animals used per group was determined to ensure a balance between statistical power, feasibility, and ethical considerations, and was approved by the relevant animal welfare authorities. To establish a subcutaneous tumor model, Panc02 cells (1 × 10⁶ in 100 μL PBS) were injected into the right flank of each C57BL/6 mouse at 6 weeks of age. Subsequent experiments were initiated when tumor volumes reached approximately 80 mm³. The maximum tumor size allowed by the ethics committee was 1500 mm³, and this limit was not exceeded during the study. All mice were housed under specific pathogen-free (SPF) conditions at 26 ± 1 °C and 50 ± 5% relative humidity with a 12-hour light/dark cycle and free access to food and water.

**Chemicals and materials**

Tetraethyl orthosilicate (TEOS), potassium permanganate (KMnO₄), sodium carbonate (Na₂CO₃), ammonia solution (25%), absolute ethanol, 2.5% glutaraldehyde, and osmium tetroxide (OsO₄) were purchased from Sinopharm Chemical Reagent Co., Ltd. (China). Phloretin (Pht), hyaluronic acid (HA), and reduced glutathione (GSH) were obtained from Shanghai Yuanye Biotechnology Co., Ltd. (China). Methylene blue (MB), bovine serum albumin (BSA), 4% paraformaldehyde, and the cystine content assay kit were provided by Beijing Solarbio Science & Technology Co., Ltd. (China). JC-1 mitochondrial membrane potential probe, DCFH-DA, Calcein-AM/PI cell viability/cytotoxicity assay kit, DAPI staining solution, immunostaining permeabilization buffer containing Triton X-100, glucose assay kit with O-toluidine, ATP assay kit, SDS-PAGE sample loading buffer, BeyoECL Plus, poly(vinylidene fluoride) (PVDF) membranes, SDS-PAGE gel quick preparation kit, BCA protein assay kit, Hoechst 33342 staining solution, fluorescent phalloidin (Actin-Tracker Green-488), and C11-BODIPY581/591 probe were all obtained from Beyotime Biotechnology Co., Ltd. (Shanghai, China). The cell counting kit (CCK-8) was purchased from Yeasen (China). The glutathione assay kit was purchased from Nanjing Jiancheng Bioengineering Institute (NJJCBIO). Erastin, Z-VAD-FMK, Necrostatin-1, Chloroquine, and Ferrostatin-1 were obtained from MedChemExpress (Shanghai, China). DeferoXamine was purchased from APExBIO Technology LLC. Dithiothreitol (DTT) was purchased from Aladdin Reagent Co., Ltd. (China). Phosphate-buffered saline (PBS) was purchased from Gibco (Shanghai, China). Mouse Panc02 cells (Catalog No. STCC20054P) were purchased from Servicebio (Wuhan Servicebio Technology Co., Ltd., China). RPMI-1640 medium was purchased from Gibco, and fetal bovine serum (FBS) was obtained from Lonsera. The G6PDH assay kit was purchased from Sangon Biotech (Shanghai, China). The WST-8-based NADP⁺/NADPH assay kit was purchased from Elabscience (China). Rabbit anti-GLUT1, anti-MYH9, anti-FLNA, anti-TLN1, anti-WAVE2, anti-NCKAP1, anti-Rac1 primary antibodies and HRP-conjugated goat anti-rabbit IgG (H+L) secondary antibody were obtained from Abclonal Technology (Wuhan, China).

**Molecular docking**

Compounds in SDF format were obtained from the PubChem database and imported into ChemDraw 3D for preliminary energy minimization using the MM2 force field. The energetically minimized conformations were saved as mol2 files. The three-dimensional structure of the target protein was obtained from the Protein Data Bank (PDB) and visualized using PyMOL. Ligand and receptor preparation, including desolvation, hydrogen addition, charge calculation, and non-polar hydrogen merging, was performed using AutoDockTools (MGLTools 1.5.6), and the prepared structures were saved as pdbqt files. Molecular docking was conducted using AutoDock Vina 1.1.2.

**Molecular dynamics (MD)**

Molecular dynamics simulations were performed using GROMACS 2025. The protein topology was generated using pdb2gmx with the AMBER14SB force field, and ligand parameters were generated based on the GAFF2 force field. The protein–ligand complex was solvated in a TIP3P water box with a minimum distance of 1.2 nm from the solute to the box edge, and Na⁺/Cl⁻ ions were added to neutralize the system and achieve an ionic strength of 0.15 M. Long-range electrostatic interactions were treated using the particle mesh Ewald (PME) method with a cutoff of 1.0 nm, and all covalent bonds involving hydrogen atoms were constrained using the LINCS algorithm. The system was first energy-minimized, followed by equilibration under the NVT and NPT ensembles. Subsequently, a 100 ns production MD simulation was performed at 310 K and 1 bar with a time step of 2 fs (50,000,000 steps). Trajectory analyses were carried out using GROMACS tools, including root mean square deviation (RMSD), root mean square fluctuation (RMSF), radius of gyration (Rg), solvent-accessible surface area (SASA), hydrogen bond analysis, and free energy landscape analysis. The binding free energy was further estimated using the MM/PBSA method implemented in the g_mmpbsa package.

**Preparation of HMnO_2_**

Typically, ethanol (10 mL), ultrapure water (5 mL), and aqueous ammonia solution (3 mL) were mixed in a beaker under magnetic stirring to obtain a homogeneous reaction medium. Tetraethyl orthosilicate (TEOS, 2 mL) was then added rapidly under vigorous stirring. The mixture was maintained at 50 °C with continuous stirring overnight to allow the formation of SiO₂ nanoparticles. The resulting SiO₂ nanoparticles were collected by centrifugation, washed thoroughly with ethanol and ultrapure water, and then redispersed for subsequent use. For MnO₂ shell deposition, the as-prepared SiO₂ nanoparticles were slowly added dropwise into an aqueous KMnO₄ solution (150 mg in 50 mL deionized water) under stirring. The mixture was further treated by ultrasonication for 6 h to facilitate uniform deposition of MnO₂ on the SiO₂ surface. After the reaction, the obtained SiO₂@MnO₂ nanoparticles were collected by centrifugation and washed with deionized water to remove residual reagents, affording a dark-brown solid. To obtain hollow mesoporous MnO₂, the SiO₂@MnO₂ nanoparticles were redispersed in an aqueous Na₂CO₃ solution and stirred at 60 °C overnight to etch the silica template. After 12 h, the product was collected by centrifugation and washed with deionized water three times to remove residual sodium silicate, yielding hollow mesoporous MnO₂ nanoparticles (HMnO₂).

**Preparation of HMnO_2_-HA**

To achieve covalent HA modification on HMnO₂, a silanization followed by carbodiimide-mediated coupling strategy was used. Briefly, HMnO₂ (10 mg) was dispersed in an ethanol/water mixture (v/v=4:1) under stirring. 3-Aminopropyltriethoxysilane (APTES, 100 μL) was added, and the mixture was stirred at 60 °C for 2 h to introduce surface amine groups. The amine-functionalized HMnO₂ (HMnO₂-NH₂) was collected by centrifugation and washed successively with ethanol and deionized water to remove unreacted APTES. Separately, hyaluronic acid (HA, 2 mg) was dissolved in 0.1 M MES buffer (5 mL, pH 5.5). 3 mg EDC and 2 mg NHS were then added, and the solution was stirred in the dark for 30 min to activate the carboxyl groups of HA. After activation, the HA solution was adjusted to pH 7.0 using PBS and immediately mixed with HMnO₂-NH₂ under continuous stirring at room temperature for 12 h to allow amide bond formation. The resulting HMnO₂-HA nanoparticles were collected by centrifugation and washed thoroughly with deionized water to remove unbound HA, and then redispersed for further use.

**Preparation of Pht@HMnO_2_-HA**

For drug loading, HMnO₂ nanoparticles (10 mg) were dispersed in a DMSO solution of phloretin (Pht, 8 mg/mL, 5 mL) and stirred overnight at room temperature to allow sufficient loading. The Pht-loaded HMnO₂ (Pht@HMnO₂) was collected by centrifugation and washed with deionized water to remove free Pht. To enable HA conjugation, Pht@HMnO₂ was redispersed in 80% ethanol (30 mL), followed by the addition of APTES (100 μL). The mixture was stirred at 60 °C for 2 h to obtain amine-functionalized Pht@HMnO₂ (Pht@HMnO₂-NH₂). The product was collected by centrifugation and washed with ethanol and deionized water. HA activation was performed as described above: HA (2 mg) was dissolved in 0.1 M MES buffer (5 mL, pH 5.5), followed by addition of EDC (3 mg) and NHS (2 mg) and stirring in the dark for 30 min. The activated HA solution was then mixed with Pht@HMnO₂-NH₂ and stirred overnight at room temperature. The final Pht@HMnO₂-HA nanoparticles were harvested by centrifugation and washed thoroughly with deionized water for further use.

**Characterization**

Transmission electron microscopy (TEM), high-angle annular dark-field scanning transmission electron microscopy (HAADF-STEM), and energy-dispersive X-ray spectroscopy (EDS) elemental mapping were performed using a JEOL JEM-2100F transmission electron microscope operating at 200 kV. The particle size distribution was analyzed by dynamic light scattering (DLS) using a Malvern Zetasizer Nano ZS90 (Malvern Instruments, UK), and the zeta potential of different samples was measured on the same Zetasizer system. UV–vis–NIR absorption spectra were recorded using a Shimadzu UV-3600 spectrophotometer. Fourier transform infrared (FT-IR) spectra were measured using a Thermo Nicolet iS50 FTIR spectrometer. X-ray photoelectron spectroscopy (XPS) analysis was conducted using a Thermo Scientific ESCALAB 250Xi spectrometer with monochromatic Al Kα radiation.

**Drug loading and encapsulation efficiency**

To evaluate the drug loading capacity of Pht@HMnO₂, a standard calibration curve of Phloretin (Pht) was generated by measuring its absorbance at ~285 nm in DMSO. After the drug loading process, the supernatant was collected by centrifugation, and the concentration of free (unloaded) Pht was determined using the same method. Drug loading (DL) and encapsulation efficiency (EE) were calculated as follows:

DL (%) = (Weight of loaded Pht / Weight of nanoparticles) × 100%

EE (%) = (Weight of loaded Pht / Total Pht added) × 100%

**In vitro Phloretin release**

For the release study, Pht@HMnO₂-HA containing an equivalent amount of Pht was dispersed in PBS under different conditions (pH 7.4 or 6.5, with or without 5 mM GSH). The suspension was sealed in a dialysis bag (MWCO: 3500 Da) and immersed in 50 mL of release medium at 37 °C with gentle shaking. At predetermined time points, 1 mL of the external solution was sampled and replaced with fresh buffer. The amount of released Pht was quantified by UV–vis spectroscopy at ~285 nm.

**pH/GSH-responsive degradation of Pht@HMnO₂‑HA**

Manganese dioxide (MnO₂) is known to be stable under neutral or basic conditions but can be reduced to Mn²⁺ in acidic or reductive environments. Particularly, MnO₂ reacts with glutathione (GSH) according to the equation:

2 GSH + MnO₂ + 2 H⁺ → GSSG + Mn²⁺ + 2 H₂O

Therefore, following the drug release experimental conditions, the morphological decomposition of Pht@HMnO₂-HA nanoparticles at different pH values (7.4 and 6.5) with or without GSH (5 mM) was observed by TEM. Samples were collected at predetermined intervals (0.5, 1, 2, and 4 h), and the TEM images were compared and analyzed.

**GSH depletion**

The consumption of glutathione (GSH) was measured using a DTNB-based assay. Briefly, to investigate concentration-dependent GSH depletion, different concentrations of Pht@HMnO₂-HA nanoparticles (0, 25, 50, 100, 150, 200 µg/mL) were incubated with GSH (1 mM) in PBS buffer (pH 6.5) at 37 °C for a fixed duration of 2 h. For time-dependent GSH depletion, a fixed nanoparticle concentration (100 µg/mL) was incubated with GSH (1 mM) under the same buffer conditions, and samples were collected at various time points (0.5, 1, 2, 4 and 6 h). After incubation, the mixtures were centrifuged (10,000 × g, 10 min), and 200 µL of the supernatant was mixed immediately with 300 µL of 2.5 mM DTNB solution. The absorbance at 412 nm was measured by a UV–vis spectrophotometer (Agilent 8453).

**Evaluation of Fenton‑like ·OH generation by MB degradation**

A methylene‑blue (MB) assay was employed to probe the ·OH‑generating capability of Pht@HMnO₂‑HA. Briefly, 1 mL reaction mixtures containing MB (20 µM), H₂O₂ (100 µM) and Pht@HMnO₂‑HA (100 µg mL⁻¹) were prepared in PBS at either pH 7.4 or pH 6.5. GSH was then introduced at final concentrations of 0, 0.25, 0.50 or 1 mM. After gentle shaking at 37 °C for a fixed period sufficient for the reaction to reach equilibrium (≈30 min), the solutions were centrifuged (10 000 × g, 5 min) and the supernatants were subjected to UV–vis scanning (400–800 nm, Agilent 8453). The decrease in the characteristic MB peak at 664 nm under different conditions—MB alone, MB + NPs, MB + H₂O₂, and MB + H₂O₂ + NPs with graded GSH—was used to compare the Fenton‑like catalytic efficiency of the nanoparticles at physiological (pH 7.4) and mildly acidic (pH 6.5) environments.

**In vitro MR imaging**

To assess the MRI imaging capability of Pht@HMnO₂‑HA and its T1-weighted signal responsiveness under GSH conditions, sample solutions with varying Mn concentrations (0.0375, 0.075, 0.15, 0.3, 0.6, and 1.2 mM) were prepared and incubated in PBS buffer at pH 7.4 (without GSH) and pH 6.5 (with 5 mM GSH) at 37 °C for 30 minutes. The samples were then collected for T1-weighted MRI scanning using a clinical 1.5 T MRI system. T1-weighted images at different Mn²⁺ concentrations were obtained, and the T1 relaxation times of each sample were measured. The longitudinal relaxivity (r₁) was calculated by plotting 1/T1 against Mn concentration to evaluate the MRI contrast performance.

**Cell culture**

Mouse Panc02 cells (Catalog No. STCC20054P) were purchased from Servicebio (Wuhan Servicebio Technology Co., Ltd., China). Cells were maintained in Roswell Park Memorial Institute 1640 medium (RPMI‑1640, Gibco) supplemented with 10 % fetal bovine serum (FBS, Lonsera) at 37 °C in a humidified incubator with 5 % CO₂.

**Cellular uptake**

Panc02 cells were seeded in confocal culture plates at a density of 5 × 10⁴ cells per well and incubated for 24 hours. Then, FITC-labeled HMnO₂ and HMnO₂-HA were respectively added to the wells and incubated with the cells for various time periods. After incubation, cells were washed three times with PBS and stained with Hoechst 33342 for 10 minutes. Finally, cellular uptake was observed using a confocal laser scanning microscope (ZEISS 880 or Leica STED).

**Cell viability and cytotoxicity assay**

Panc02 cells were seeded into 96-well plates at a density of 5,000 cells per well. After 24 hours of incubation, the culture medium was replaced with fresh medium containing different treatment conditions. For the cytotoxicity assay, cells were treated with different concentrations of HMnO₂-HA, Pht, or Pht@HMnO₂-HA for 12 hours. For the rescue experiments, cells were pretreated with Z-VAD-FMK (10 μM), Nec-1 (10 μM), CQ (10 μM), Fer-1 (10 μM), DFO (100 μM), TCEP (1 mM), or DTT (1 mM) for 1 hour, followed by treatment with Pht@HMnO₂-HA for another 12 hours. After treatment, each well was replaced with 100 μL of fresh medium containing 10% CCK-8 reagent. After incubation for 1 hour at 37 °C, the absorbance was measured at 450 nm using a microplate reader. Cell viability was calculated using the following formula: Cell viability (%) = [(Absorbance of treatment group − Absorbance of blank) / (Absorbance of control group − Absorbance of blank)] × 100.

**Live/Dead cell staining assay**

For live/dead cell staining assay, Panc02 cells were seeded into 48-well plates at a density of 4 × 10⁴ cells per well and incubated for 12 hours under different treatment conditions. After incubation, the culture medium was removed and the cells were gently washed with PBS. Then, 200 μL of Calcein-AM/PI staining working solution (prepared according to the manufacturer’s instructions) was added to each well. Cells were incubated at 37 °C for 30 minutes in the dark, and then observed and imaged using a fluorescence microscope. Green fluorescence (Calcein-AM) indicates live cells, while red fluorescence (PI) indicates dead cells.

**Intracellular ROS detection**

Panc02 cells were seeded in confocal dishes at a density of 5 × 10^4^ cells per dish and incubated for 24 hours. Cells were then treated with different formulations for another 12 hours. After the treatment, cells were washed three times with PBS and incubated with 10 μM DCFH-DA working solution at 37 °C for 20 minutes in the dark. Following incubation, excess dye was removed by washing with PBS, and cells were observed using a confocal microscopy (IX71, Olympus).

**Lipid peroxidation assay**

Panc02 cells were seeded in confocal dishes at a density of 5 × 10⁴ cells per dish and incubated for 24 h. The cells were then treated with various formulations for another 12 h. After washing three times with PBS, the cells were stained with BODIPY™ 581/591 C11 probe according to the manufacturer’s instructions and incubated at 37 °C for 30 minutes in the dark. Subsequently, the stained cells were imaged by a CLSM (IX71, Olympus). Lipid peroxidation levels were evaluated by the shift from red (reduced form) to green (oxidized form) fluorescence.

**JC-1 assay**

Panc02 cells were incubated in confocal culture plates (5 × 10^4^ cells/plate) for 24 h, followed by incubation with different formulations for 12 h. After three times washing with PBS, the cells were treated with a JC-1 assay kit to analyze the mitochondrial membrane potential via confocal microscopy (IX71, Olympus).

**Bio-TEM**

Panc02 cells were grown to ~80 % confluence, exposed to the indicated treatments for 24 h, and fixed in 2.5 % glutaraldehyde (4 °C, overnight). After PBS washes, cells were post‑fixed with 1 % OsO₄, dehydrated through graded ethanol, and embedded in epoxy resin. Ultrathin sections (≈ 70–90 nm) were collected on copper grids and examined at 200 kV with a bio‑TEM to evaluate mitochondrial alterations such as swelling, cristae disruption, and vacuolization.

**Intracellular ATP assay**

ATP levels in Panc02 cells were assessed using an ATP assay kit (Beyotime, China) following the manufacturer’s protocol. Briefly, 5,000 cells were plated into 96-well plates and incubated for 24 h. After treatment with different formulations, 100 μL of freshly prepared detection reagent was added to each well, and the plate was shaken gently at room temperature for 10 min. Luminescence was then recorded using a microplate reader (BioTek, Gen5).

**Intracellular glucose assay**

Panc02 cells were seeded in six-well plates (2 × 10⁵ cells/well) and cultured for 24 h, followed by treatment with different formulations for 12 h. After washing with PBS three times, intracellular glucose levels were measured using a glucose assay kit (Beyotime, China) according to the manufacturer’s protocol.

**Intracellular G6PDH activity assay**

Panc02 cells (2 × 10⁵ cells/well) were treated with various formulations for 12 h. Cells were then washed with PBS and lysed, and G6PDH enzymatic activity was determined using a G6PDH assay kit (Sangon Biotech, Shanghai) following the kit instructions.

**Intracellular NADP⁺/NADPH assay**

After 24 h of treatment with the indicated formulations, Panc02 cells were harvested and washed with PBS. Intracellular NADP⁺/NADPH ratios were quantified using a WST-8-based NADP⁺/NADPH assay kit (Elabscience, China) according to the manufacturer’s instructions.

**Intracellular Cystine and Cysteine assay**

For free cysteine determination, treated cells were lysed and the supernatant was mixed with denaturing and reducing buffers according to the kit (Solarbio, Beijing) protocol to convert disulfide bonds into free thiols. After two rounds of protein precipitation, washing, and resuspension, Ellman’s reagent was added for color development, and the absorbance was recorded at 412 nm. Cysteine levels were calculated from a GSH calibration curve. For cystine measurement, a parallel sample processed without the reducing buffer served as a control; the difference between the reduced and non‑reduced samples was used to indirectly quantify cystine.

**Fluorescent staining of actin filaments**

Panc02 cells (5 × 10^4^ cells per well) were seeded onto 12 mm glass coverslips and cultured until reaching 60%–70% confluence, followed by different treatments for 24 h. After PBS washing, cells were fixed with 4% paraformaldehyde for 15 min and permeabilized with 0.1% Triton X-100 for 10 min. Then, cells were incubated with fluorescent phalloidin (1:1000 dilution) for 1 h at room temperature in the dark, followed by nuclear staining with DAPI (1 μg/mL) for 10 min. Finally, F-Actin morphology was observed using confocal microscopy.

**RNA sequencing**

Cells were seeded into 6-well plates and cultured for 24 hours, followed by different treatments: control group and Pht@HMnO₂‑HA treatment group. After 24 hours of intervention, the cells were collected and subjected to RNA sequencing according to the requirements of Majorbio Bio-Pharm Technology Co., Ltd. (Shanghai, China).

**Western blotting**

Total proteins were extracted from Panc02 cells and mouse tumor tissues after different treatments. Cells were lysed after PBS washing, while tissues were ground in liquid nitrogen before lysis. Protein concentrations were determined using the BCA assay. Protein samples were divided into two parts and mixed separately with reducing and non-reducing loading buffer, then heated at 100 °C for 5 minutes. Proteins were separated on 6% or 10% SDS-PAGE gels and transferred onto PVDF membranes. After blocking with 5% skim milk for 1 hour, membranes were incubated overnight at 4 °C with primary antibodies (NCKAP1, WAVE2, MYH9), followed by incubation with HRP-conjugated secondary antibodies for 1 hour at room temperature. After washing with TBST, the membranes were developed using ECL detection reagents. Protein bands were imaged with a gel imaging system, and band intensities were quantified using ImageJ software, normalized to β-Actin or Vinculin as loading controls.

**In vivo MR imaging**

First, tumor model mice were built by injecting 100 μL of Panc02 cells (1 × 10^6^ cells/mL). After 10 days, the volume was about 100 mm^3^, and a GE SIGNA Premier 3.0T MRI scanner was employed to evaluate the in vivo MR imaging performance of Pht@HMnO₂‑HA. Before injection, the T1-weighted MR imaging of the tumor site was recorded. Then, the mouse was injected with 100 μL of Pht@HMnO₂‑HA solution (1 mg/mL) via a tail vein. After 0, 2, 4, 8 h, the T1-weighted MR imaging was re-recorded.

**In vivo antitumor efficiency**

Tumor‑bearing mice were randomly divided into four groups (n = 5 per group): Control, HMnO₂‑HA, Pht, and Pht@HMnO₂‑HA. On days 0, 3, 6, 9 and 12, the Control group received intravenous PBS, whereas the other groups were given the corresponding formulations via tail‑vein injection. Body weight and tumor dimensions were recorded every second day; tumor volume (V) was calculated as V = l × w² / 2, where l and w denote tumor length and width, respectively. Mice were euthanized on day 15, and tumors together with major organs (heart, liver, spleen, lung, kidney) were harvested. Tumor sections were subjected to H&E, TUNEL, Ki67, GLUT1, and WAVE2 staining, while the major organs were examined by H&E staining to assess systemic toxicity.

**Hemolysis analysis of Pht@HMnO₂‑HA**

Mouse blood was collected from the supraorbital veins and centrifuged at 1000 × g for 10 minutes to remove the serum. The remaining blood cells were resuspended in saline and washed twice with saline. The cells were then incubated with different concentrations of Pht@HMnO₂‑HA for 30 minutes. After incubation, the mixtures were centrifuged at 3000 × g for 5 minutes, and the absorbance of the supernatant was measured at 405 nm.

**Biochemistry parameter analysis**

16 male C57BL/6 mice (6 weeks old) were randomly assigned to four groups and administered different treatments. On day 14 post-treatment, blood samples were collected, and serum was obtained by centrifugation at 1000 × g for 5 minutes and kept at 4 °C. Various serum biochemical markers, including AST, ALT, ALP, TBil, DBil, IBil, UREA and CREA, were measured using commercial assay kits according to the manufacturer’s instructions.

**Statistical analysis**

All experiments were repeated at least three times. Quantitative data were indicated as mean ± S.D. The software of GraphPad Prism 9.0 was adopted to assess the statistical analysis, which was performed using the Student’s t-test and one/two-way ANOVA. The statistical significance was attained at *p < 0.05, **p < 0.01, ***p < 0.001, and ****p < 0.0001.

**Figure S1.** Pan-cancer expression profile of SLC7A11 across TCGA tumor types. Gene expression levels in tumor and corresponding normal tissues are shown as log (TPM + 1). Statistical significance between groups is indicated.

**Figure S2.** Kaplan–Meier analysis of overall survival in PAAD patients stratified by SLC7A11 expression level. Patients were divided into high- and low-expression groups based on median expression. Differences in survival were evaluated using the log-rank test.

**Figure S3.** Pan-cancer expression profile of SLC2A1 (GLUT1) across TCGA tumor types. Gene expression levels in tumor and corresponding normal tissues are presented as log (TPM + 1). Statistical significance between groups is indicated.

**Figure S4. Molecular dynamics analysis of the phloretin–GLUT1 complex.** (A) Root Mean Square Fluctuation (RMSF) of GLUT1 residues during the 100 ns molecular dynamics simulation. (B) Time evolution of Solvent-Accessible Surface Area (SASA) of the protein–ligand complex over the simulation period. (C) Per-residue binding free energy decomposition of key residues contributing to the interaction between phloretin and GLUT1. (D) Free energy landscape of the phloretin–GLUT1 complex.

**Figure S5. Molecular docking analysis of hyaluronic acid (HA) with CD44.** (A) Molecular docking conformation of HA with CD44. Cyan ribbon: CD44; magenta sticks: HA. (B) Close-up view of the binding site showing interactions between HA and key residues of CD44. (C) Two-dimensional interaction diagram of HA with representative residues of CD44.

**Figure S6. Structural characterization of the nanoparticles.** (A) Transmission electron microscopy (TEM) image of HMnO2. (B) Transmission electron microscopy (TEM) image of Pht@HMnO2-HA. (C) Energy-dispersive X-ray spectroscopy (EDS) spectrum of Pht@HMnO2-HA.

**Figure S7.** Hydrodynamic diameter (DLS) of Pht@HMnO₂-HA dispersed in H₂O, PBS, and DMEM over 7 days. Data are presented as mean ± SD.

**Figure S8.** X-ray photoelectron spectroscopy (XPS) survey spectrum of HMnO_2_.

**Figure S9.** X-ray photoelectron spectroscopy (XPS) spectra of the C1s region of HMnO_2_.

**Figure S10.** X-ray photoelectron spectroscopy (XPS) spectra of the Mn2p region of HMnO_2_.

**Figure S11.** (A) Drug loading content (DL) and encapsulation efficiency (EE) of phloretin (Pht) in Pht@HMnO₂ nanoparticles. (B) Cumulative Pht release from Pht@HMnO₂-HA under pH 7.4 without GSH and pH 6.5 with 5 mM GSH at 37 °C. Data are presented as mean ± SD.

**Figure S12.** In vitro T1-weighted MR images of Pht@HMnO₂-HA suspensions with increasing Mn concentrations (0.0375–1.2 mM) acquired under pH 7.4 without GSH and pH 6.5 with GSH.

**Figure S13.** Cell viability of cells treated with HMnO₂-HA (A), free phloretin (Pht) (B), and Pht@HMnO₂-HA (C) at different concentrations.

**Figure S14.** Quantitative analysis of intracellular total glutathione and GSSG/GSH levels under different treatment conditions. Data are presented as mean ± standard deviation (SD). Statistical significance: *p < 0.05, **p < 0.01, ***p < 0.001, and ****p < 0.0001; ns, not significant.

**Figure S15.** Fluorescence microscopy images and quantitative analysis of intracellular lipid peroxidation (LPO) in cells treated with Control, HMnO₂-HA, Pht, and Pht@HMnO₂-HA. Nuclei were stained with Hoechst (blue), while the oxidized and reduced forms of the LPO-sensitive probe are shown in green and red, respectively. Merged images are shown in the bottom row. Data are presented as mean ± standard deviation (SD). Statistical significance: *p < 0.05, **p < 0.01, ***p < 0.001, and ****p < 0.0001; ns, not significant.

**Figure S16.** Fluorescence microscopy images and quantitative analysis of mitochondrial membrane potential (ΔΨm) in cells treated with Control, HMnO₂-HA, Pht, and Pht@HMnO₂-HA. Red fluorescence corresponds to JC-1 aggregates (high ΔΨm), while green fluorescence corresponds to JC-1 monomers (low ΔΨm). Data are presented as mean ± standard deviation (SD). Statistical significance: *p < 0.05, **p < 0.01, ***p < 0.001, and ****p < 0.0001; ns, not significant.

**Figure S17.** Cell viability of Panc02 cells treated with Pht@HMnO₂-HA in the presence of Z-VAD-FMK (10 μM), Nec-1 (10 μM), CQ (10 μM), Fer-1 (10 μM), DFO (100 μM), TCEP (1 mM), or DTT (1 mM) for 12 h. Data are presented as mean ± standard deviation (SD). Statistical significance: *p < 0.05, **p < 0.01, ***p < 0.001, and ****p < 0.0001; ns, not significant.

**Figure S18.** Gene Ontology (GO) annotations analysis (Level 2) of differentially expressed genes between the Pht@HMnO₂-HA group and the control group. The GO terms are classified into biological process, cellular component, and molecular function, and the number of genes associated with each term is shown.

**Figure S19.** KEGG pathway annotations analysis of differentially expressed genes between the Pht@HMnO₂-HA group and the control group. Pathways are categorized into environmental information processing, organismal systems, and human diseases, and the number of genes annotated to each pathway is indicated.

**Figure S20.** Reactome pathway annotation analysis of differentially expressed genes between the Pht@HMnO₂-HA group and the control group. The number of genes associated with each Reactome pathway is shown.

**Figure S21.** Gene set enrichment analysis (GSEA) based on the entire transcriptome comparing the Pht@HMnO₂-HA group and the control group. The normalized enrichment score (NES) is shown on the x-axis. Bubble size represents the number of genes in each gene set, and color indicates the adjusted P value (Padjust).

**Figure S22.** Representative GSEA enrichment plots of selected metabolic and functional pathways in the Pht@HMnO₂-HA versus control comparison, including Carbon metabolism (A), Citrate cycle (TCA cycle) (B), antioxidant activity (C), and Apoptosis (D). Enrichment score (ES), normalized enrichment score (NES), and gene rank positions are indicated.

**Figure S23.** Hemolysis evaluation of HMnO₂-HA and Pht@HMnO₂-HA. (A) Representative photographs of red blood cells after incubation with HMnO₂-HA (1) and Pht@HMnO₂-HA (2) at different concentrations. (B) Quantitative analysis of hemolysis ratios corresponding to the samples shown in (A).

**Figure S24.** Blood biochemical analysis in mice after different treatments. (A) Alanine aminotransferase (ALT), (B) alkaline phosphatase (ALP), (C) aspartate aminotransferase (AST), (D) total bilirubin (TBIL), (E) direct bilirubin (DBIL), and (F) indirect bilirubin (IBIL) levels in serum collected from mice treated with Control, HMnO₂-HA, Pht, and Pht@HMnO₂-HA. Data are presented as mean ± standard deviation (SD). Statistical significance: *p < 0.05, **p < 0.01, ***p < 0.001, and ****p < 0.0001; ns, not significant.

**Figure S25.** Immunofluorescence staining of NCKAP1 in tumor tissues treated with Control, HMnO₂-HA, Pht, and Pht@HMnO₂-HA.

**Figure S26.** Immunofluorescence staining of Rac1 in tumor tissues treated with Control, HMnO₂-HA, Pht, and Pht@HMnO₂-HA.

**Figure S27.** Immunofluorescence staining of GPX4 in tumor tissues treated with Control, HMnO₂-HA, Pht, and Pht@HMnO₂-HA.
